# CISRMamba: Cross-Modal Interaction and Scan-Routing Mamba for Multi-Sensor Flood Inundation Mapping

**DOI:** 10.3390/s26165224

**Published:** 2026-08-18

**Authors:** Haoran Feng, Chenyang Xiao, Ruiyang Lin, Yuxuan Chen, Linxing Liang, Bin Lin

**Affiliations:** 1 College of Photonic and Electronic Engineering, Fujian Normal University, Qishan Campus, 1 Keji Road, University Town, Fuzhou 350117, China; 136132023058@student.fjnu.edu.cn (H.F.); 136132023008@student.fjnu.edu.cn (R.L.); 136132023056@student.fjnu.edu.cn (Y.C.); 136132023036@student.fjnu.edu.cn (L.L.); 2 School of Computer Science and Technology, Beijing Institute of Technology, No. 5 South Zhongguancun Street, Haidian District, Beijing 100081, China; 1120230958@bit.edu.cn

**Keywords:** flood mapping, optical–SAR fusion, multimodal fusion, state space models, Mamba, boundary refinement

## Abstract

Accurate flood mapping is essential for disaster response, yet optical–SAR fusion remains difficult when clouds, speckle noise, and terrain interference cause the two sensors to provide uneven or conflicting evidence. Multimodal models may suffer from “modality collapse”, while fixed scan patterns make water boundaries difficult to delineate. To address these issues, we propose CISRMamba, a multimodal state space model (SSM) based on a lightweight MobileMamba backbone. A Deformable Scan Router (DSR) adapts the scan trajectory to irregular flood contours while preserving SSM efficiency. Instead of forcing all regions into a rigid “align-then-fuse” process, the Feature Complementary Module (FCM) suppresses discrepant responses, supplies complementary information where one modality is incomplete, or enhances consistent evidence according to the local optical–SAR relationship. The Refined Feature Integration (RFI) module reduces distributional shift and modality collapse through a difference-guided residual, while the Spectral Refinement Block (SRB) recovers high-frequency spatial details weakened by downsampling. Experiments on CAU-Flood and Wuhan show that CISRMamba achieves mIoU scores of 91.53% and 58.67%, respectively, outperforming baselines based on CNNs, Transformers, and Mamba. These results indicate that CISRMamba combines context modeling with boundary-level refinement for efficient flood monitoring under heterogeneous weather and scene conditions.

## 1. Introduction

In recent years, global climate change and rapid urbanization have made floods among the most frequent and destructive natural disasters worldwide, resulting in significant economic losses and persistent risks to both human populations and the environment [1]. Consequently, rapid, accurate, and comprehensive mapping of flooded areas is essential to support emergency response, minimize damage, facilitate recovery, and assess long-term risks [2]. Earth-observation remote sensing provides a practical means of monitoring flood-related surface changes over large areas and extended periods [3,4,5]. When observations from several satellite sensors are considered together, they describe flood-prone terrain more completely than conventional ground surveys can.

Early flood-monitoring studies combined dedicated sensing systems, hydrological models, and hand-crafted image statistics [6,7,8,9]. These efforts were followed by automated image-based methods for high-resolution SAR observations [10,11]. Most of this work nevertheless focused on a single satellite modality. For optical imagery, McFeeters [12] introduced the Normalized Difference Water Index (NDWI), which became a standard index for water-body extraction. In the SAR domain, Otsu’s automated histogram thresholding method [13] offered an early basis for separating water from land. Bovolo and Bruzzone [14] subsequently extended the Change Vector Analysis (CVA) framework for unsupervised change detection. Classical learning methods followed these statistical approaches, among them Bayesian networks [15] and Support Vector Machines (SVMs) [16]. Advances in deep learning subsequently extended image-based flood analysis across aerial and satellite observations [17,18]. For example, Nemni et al. [18] used Fully Convolutional Networks (FCNs) for end-to-end modeling of SAR imagery, making rapid post-disaster mapping more efficient.

However, the physical limits of a single sensor still constrain these methods. Optical sensors capture detailed spectral information for visual interpretation, but usable observations depend on weather conditions [19] and are often blocked by the dense cloud cover that accompanies severe floods. Synthetic Aperture Radar (SAR), by contrast, provides active sensing by day or night and observes through clouds and precipitation [20]. However, speckle noise and geometric distortion in SAR images make precise boundary extraction difficult [21]. Flood mapping is therefore both a segmentation problem and a heterogeneous-data processing problem: complementary physical measurements must be combined without removing evidence that is specific to either sensor. Consequently, methods that use handcrafted features or a single modality cannot make full use of the complementary evidence present in complex flood scenes.

In recent years, deep multimodal networks have been used to draw on the different strengths of individual sensors. Their architectural development can be viewed in three stages. Initially, convolutional neural networks (CNNs) formed the dominant approach. Early multi-stream models such as FC-Siam-conc [22] and SNUNet [23] combined modality-specific representations through direct concatenation or dense skip connections. However, channel stacking alone does not address the radiometric difference between optical reflectance and SAR backscatter. Later CNN frameworks introduced more structured forms of feature interaction. DSA-Net applies deep supervision and attention to bitemporal change features [24]; heterogeneous optical–SAR methods employ gated cross-modal attention in CMCDNet [25], asymmetric semantic alignment in ASANet [26], and multilevel feature fusion in MFFNet [27]. Despite these improvements, the local receptive fields of CNN architectures [28,29] limit access to the broad spatial context of large flood areas with complex topology. To capture these long-range dependencies, Vision Transformers (ViTs) were introduced in the second stage. BIT [30] and ChangeFormer [31], for example, use self-attention to represent global context at several levels and supply non-local cues for extracting continuous water bodies. Nevertheless, their quadratic computational cost is a major limitation when high-resolution satellite images are processed at scale [32,33]. As a result, the third stage introduced state space models (SSMs) to address this cost. Based on the Mamba architecture [34], visual models such as RSMamba [35], ChangeMamba [36], and SD-Mamba [37] provide global receptive fields with linear computational complexity, which makes them suitable for large-scale Earth observation. In rapid flood mapping, Li et al. [5] developed UAFNet and UADPNet for SAR and optical imagery, respectively, integrating multiscale representation learning, uncertainty-guided feature refinement, and model ensembling.

Among recent optical–SAR methods, CMCDNet combines gated fusion with cross-modal self-attention to enhance multistage features [25], whereas M2CD uses feature–expert similarity to route features to modality-specific top-*k* convolutional experts and introduces optical-to-SAR self-distillation [38]. In contrast, FCM associates pixel-wise responses for discrepancy, complementation, and consistency with distinct interaction operations. RFI retains the signed optical–SAR difference as a separate residual alongside spatial fusion and channel recalibration. This design also differs from FC-Siam-diff, which provides the decoder with the absolute feature difference as a skip input [22].

Related deformable-scanning and wavelet-domain strategies are exemplified by DefMamba [39] and WaveNet [40], respectively. CISRMamba incorporates these principles at different stages of the flood-mapping pipeline: DSR places a learned spatial warp and an approximate inverse warp around the unchanged SS2D operator, whereas SRB refines the low- and high-frequency components of the fused decoder output before reconstruction.

**Figure 1:**
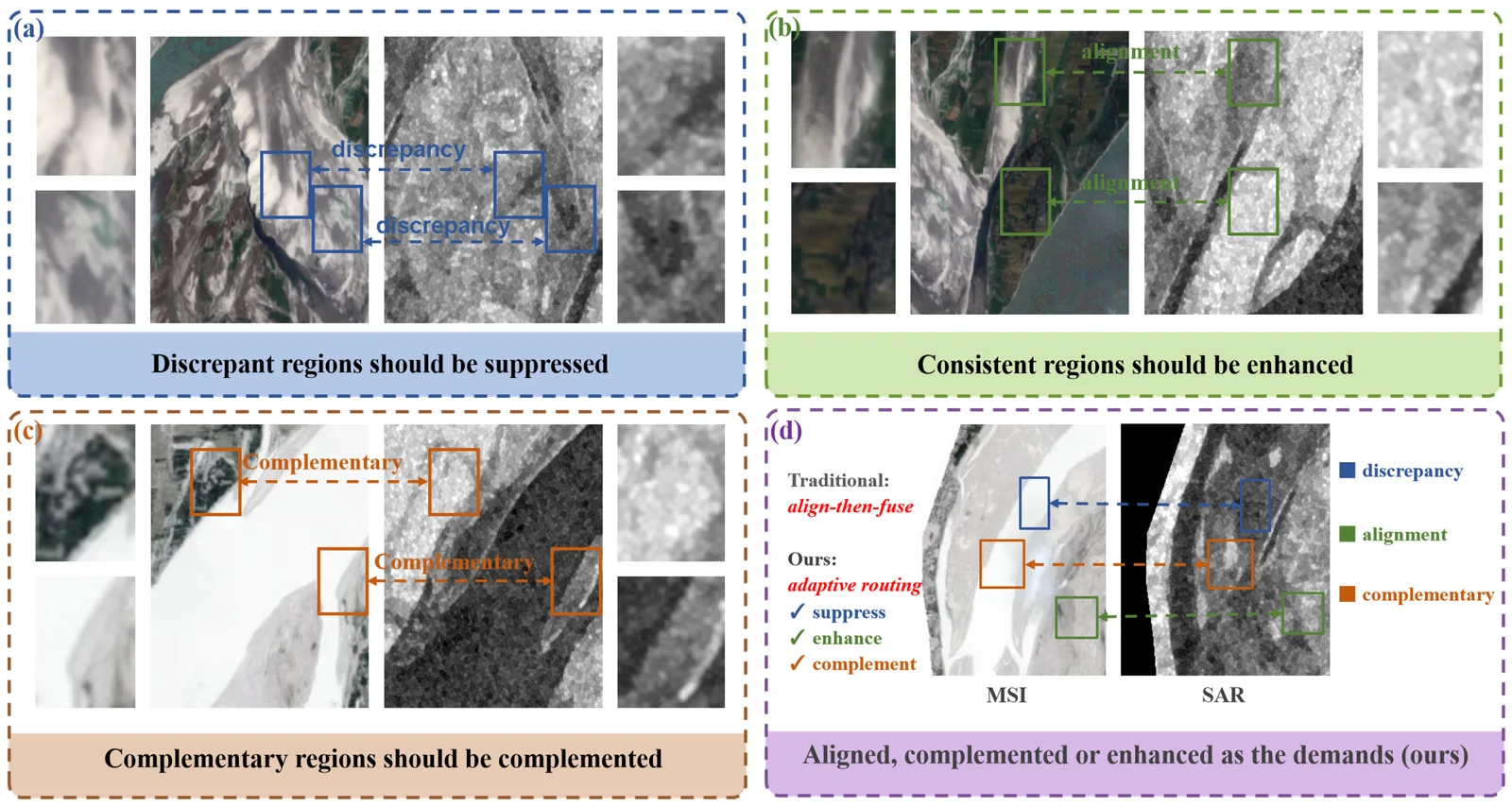
The relationship between optical Multispectral Instrument (MSI) imagery and SAR data changes across a scene. The boxes illustrate three common cases: (**a**) discrepant regions, in which the sensors return conflicting or noisy responses; (**b**) consistent regions, in which their responses largely agree; and (**c**) complementary regions, in which one modality supplies information missed by the other. Panel (**d**) summarizes the region-dependent behavior implemented by FCM: features are aligned where cross-modal correspondence is reliable, complemented where one modality supplies missing information, and enhanced where mutually consistent evidence can be reinforced, while unreliable discrepant responses are suppressed.

Despite recent advances, three main bottlenecks remain in multimodal flood mapping. First, in early direct-fusion schemes, interaction can carry noise from one modality into the other. Subsequent “align-then-fuse” methods perform semantic alignment before integration; however, a rigid transformation can discard complementary information. The relationship between optical and SAR modalities is inherently heterogeneous and spatially variable. As shown in Figure 1, local cross-modal contexts can be categorized into three types: discrepant regions, where the sensors produce conflicting or noise-corrupted responses (Figure 1a); consistent regions, where both modalities agree and reinforce each other (Figure 1b); and complementary regions, where one modality provides information absent in the other, such as SAR penetrating cloud-occluded optical imagery (Figure 1c). Since one scene can contain all three contexts, uniform alignment before fusion is often suboptimal. Figure 1d summarizes the region-dependent operation implemented by FCM: it aligns features where cross-modal correspondence is reliable, complements them where one modality supplies missing information, and enhances them where mutually consistent evidence can be reinforced, while suppressing unreliable discrepant responses.

Second, standard fusion architectures face challenges in addressing shifts in feature distributions arising from the differing imaging mechanisms of optical and SAR data. This often results in “modality collapse”: the network places disproportionate weight on optical features and makes little use of the SAR branch, particularly when dense clouds obscure the optical signal. Third, an additional bottleneck lies in the rigid spatial trajectories used by existing state space models (SSMs). Because these scans generally follow rectilinear paths, they do not match the irregular, curved boundaries of natural floodwaters and consequently blur semantic information near the edges.

To overcome these limitations, this paper presents CISRMamba, an efficient multimodal state space model for optical–SAR flood mapping. Cross-modal interaction forms the main path of the model. Within this path, the Feature Complementary Module (FCM) adapts feature complementation to local conditions, while Refined Feature Integration (RFI) balances the optical and SAR contributions through a difference-guided residual. Two auxiliary modules support the fusion process: the Deformable Scan Router (DSR) provides boundary-aware scanning, and the Spectral Refinement Block (SRB) restores high-frequency spatial detail. The contributions are as follows:

1. Region-Dependent Cross-Modal Fusion. As the primary fusion component, FCM evaluates the local optical–SAR relationship. It responds separately to discrepant, consistent, and complementary regions, replacing rigid align-then-fuse alignment with context-dependent interaction.
2. Difference-Guided Feature Integration. RFI forms the second core fusion component. A cross-modal difference residual is inserted into the integration path to reduce modality collapse and retain useful SAR contributions when optical observations are degraded.
3. Auxiliary Boundary-Aware Scanning. DSR provides an auxiliary structural component that predicts local offsets. These offsets allow the scan trajectory to follow irregular flood boundaries more closely and reduce semantic ambiguity near water–land edges.
4. Auxiliary Spatial Detail Refinement. SRB provides the auxiliary refinement stage. It uses the discrete wavelet transform (DWT) to recover high-frequency boundary cues for finer inundation delineation.

CISRMamba is evaluated on the Wuhan and CAU-Flood flood-segmentation benchmarks and outperforms the existing baselines on both datasets. The experiments show that FCM/RFI fusion accounts for the main improvements, while DSR and SRB provide further boundary refinement.

## 2. Methods

### 2.1. Overall Architecture of CISRMamba

**Figure 2:**
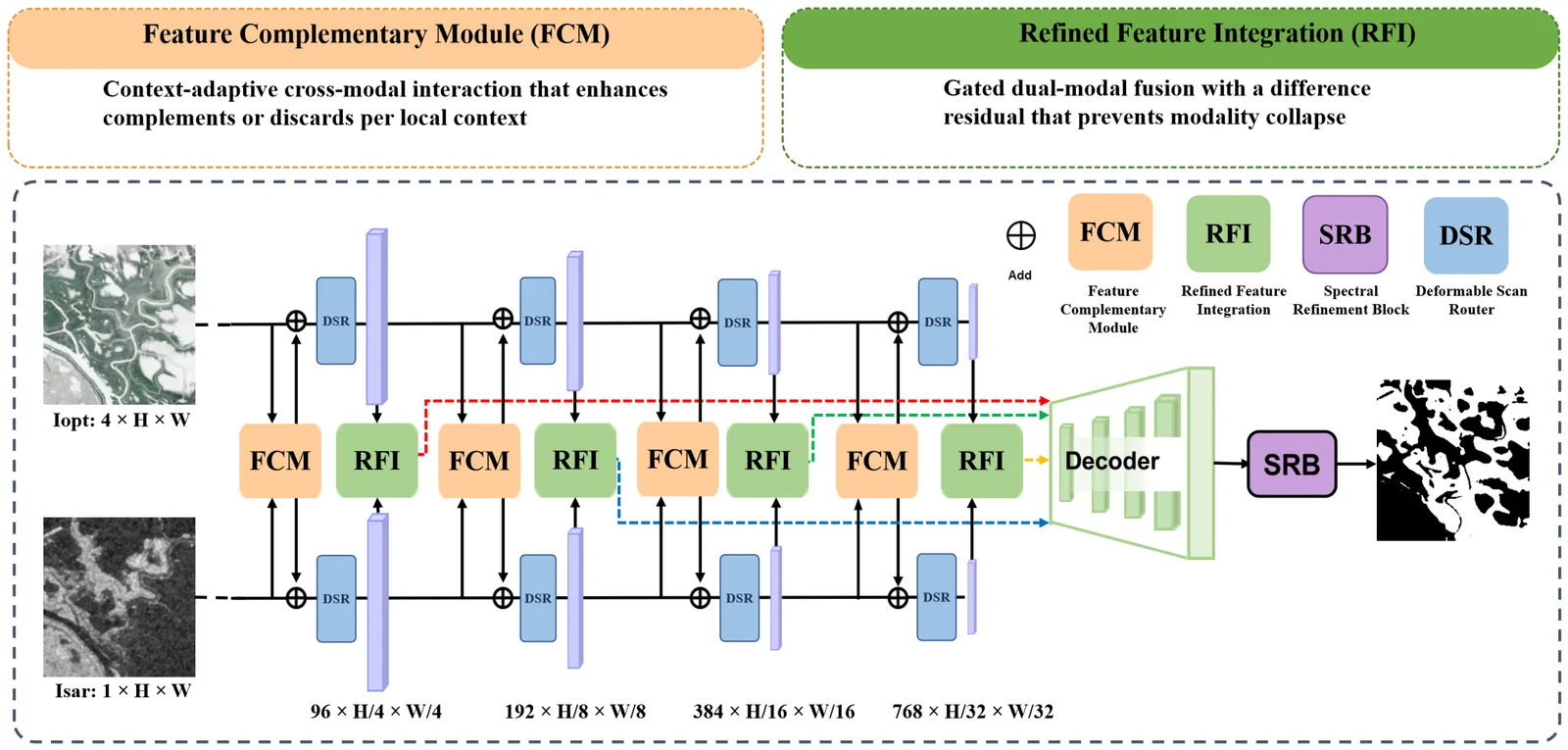
Overall architecture of CISRMamba. A dual-branch backbone encodes the optical and SAR inputs over four stages, each preceded by a Deformable Scan Router (DSR). The Feature Complementary Module (FCM) and Refined Feature Integration (RFI) blocks fuse the two modalities at every scale, and a progressive decoder with a final Spectral Refinement Block (SRB) produces the flood map. The top panels summarize FCM and RFI. Solid arrows denote feature propagation, and colored dashed arrows denote multiscale connections to the decoder.

Figure 2 shows the architecture of CISRMamba. Its inputs are a co-registered optical image $I_{\mathrm{opt}}\in R^{H\times W\times 4}$ and a SAR image $I_{\mathrm{sar}}\in R^{H\times W\times 1}$; the output is a binary flood map $\hat{Y}\in R^{H\times W}$. Processing proceeds through dual-branch encoding, cross-modal interaction, and progressive decoding. The optical and SAR encoders, $E_{\mathrm{opt}}$ and $E_{\mathrm{sar}}$, share the same structure but have separate parameters. Both use a four-stage lightweight Mamba backbone with wavelet-enhanced SS2D. Before each scan, the Deformable Scan Router (DSR) warps the tokens so that the scan follows irregular flood boundaries more closely. The convolutional stem (stride 4) and subsequent patch-merging layers produce ${\left\{F_{\mathrm{opt}}^{l}\right\}}_{l=1}^{4}$ and ${\left\{F_{\mathrm{sar}}^{l}\right\}}_{l=1}^{4}$ at strides {4, 8, 16, 32}, with {96, 192, 384, 768} channels.

For each scale *l*, the two encoder streams pass through separate convolutional heads, both producing features with d=64 channels. Fusion is determined for individual locations rather than by one fixed alignment operation. FCM makes this decision with a learned three-way gate that selects enhancement, complementation, or discrepancy suppression. The routed features then enter RFI, where spatial gating, channel recalibration, and a cross-modal difference residual produce $f^{l}$. Performing these steps at all four scales yields the single-stream pyramid ${\left\{f^{l}\right\}}_{l=1}^{4}$.

The decoder traverses the fused pyramid from top to bottom. At each level, content-aware alignment corrects the spatial mismatch with the adjacent feature, after which a channel-enhanced residual block performs refinement. At the final stage, SRB recovers high-frequency boundary information weakened by repeated downsampling. Finally, bilinear upsampling brings the prediction back to the input resolution.

### 2.2. Mechanistic Rationale of Module Coupling

The four modules are placed at different points along one processing path. Before fusion, FCM examines the available evidence at each location. Optical and Synthetic Aperture Radar (SAR) observations may repeat the same information in one region, complement each other in another, or become unreliable because of clouds, speckle, or background noise. Consequently, FCM directs the local responses to enhancement, complementation, or discrepancy suppression. This routing passes useful evidence between the modalities while filtering unreliable signals.

Refined Feature Integration (RFI) receives the two streams after FCM and keeps their contributions balanced. Without this step, a dual-stream network can favor whichever input is easier to optimize, such as clear optical imagery or SAR observations when clouds obscure the scene. RFI retains the difference between the optical and SAR features so that the output continues to use both sources instead of being controlled by one of them.

These four modules address distinct limitations at different stages of the network. FCM and RFI focus on multimodal optical–SAR fusion: FCM regulates region-dependent information exchange, whereas RFI integrates the two streams while retaining their modality-specific differences. DSR and SRB address limitations of the visual Mamba pathway. Because standard rectilinear scans cannot follow curved or fragmented flood boundaries, DSR rearranges the features before the SSM scan to align contextual propagation more closely with local flood geometry. Repeated encoding, fusion, and downsampling can also weaken high-frequency water–land boundary cues; SRB refines these cues at the decoder output. Thus, each module addresses a specific fusion or backbone limitation rather than simply increasing network depth.

### 2.3. Dual-Encoder

Optical–SAR flood detection depends on global context; however, full self-attention becomes computationally expensive at high image resolutions. CISRMamba therefore uses Mamba, whose discrete state space models (SSMs) [34] capture long-range dependencies with linear computational complexity. After zero-order-hold discretization, the SSM maps an input token $x_{k}$ to an output $y_{k}$ through the hidden state $h_{k}$ at scan step *k*. In Mamba, $\bar{A}=\exp\left(\Delta A\right)$, $\bar{B}$, and *C* depend on the input, so information is propagated selectively:

(1) $$h_{k}=\bar{A}h_{k-1}+\bar{B}x_{k},\;y_{k}=Ch_{k}+Dx_{k}$$

where the discretization step Δ maps the continuous parameters (A,B) to their discrete counterparts, whereas the feed-through term *D* passes the input directly to the output. CISRMamba builds its hierarchical feature pyramid with a modified MobileMamba [41] backbone. Applying Mamba to optical–SAR flood imagery requires addressing three issues: adapting its one-dimensional state recurrence to a two-dimensional grid, separating regional structure from boundary detail rather than processing both along the same scan path, and accommodating irregular flood shapes that fixed rectilinear scans cannot follow. Mamba models long-range dependencies with linear complexity, but its state recurrence is inherently one-dimensional, whereas remote-sensing features lie on a two-dimensional grid. SS2D bridges this dimensional mismatch by unfolding an H×W feature map in horizontal, reverse-horizontal, vertical, and reverse-vertical orders. After selective state propagation along each sequence, the outputs are restored to their original two-dimensional positions, allowing every location to aggregate long-range context from multiple directions. However, standard SS2D still processes different frequency components along the same scan path and combines the four fixed directions without accounting for their scene-dependent importance.

At each stage, a lightweight Mamba block receives $X\in R^{B\times C\times H\times W}$, where *B*, *C*, *H*, and *W* denote the batch size, channel count, height, and width. The block first applies a residual depthwise convolution DWConv(·) and then a feed-forward network FFN(·), yielding the intermediate feature $X^{\prime }$:

(2) $$X^{\prime }=\mathrm{FFN}\left(\mathrm{DWConv}(X)+X\right)+\mathrm{DWConv}\left(X\right)+X$$

The Mixer partitions $X^{\prime }$ into the global, local, and identity splits $X_{g}$, $X_{l}$, and $X_{\mathrm{id}}$, respectively, so that $X^{\prime }=[X_{g}\|X_{l}\left\|X_{\mathrm{id}}\right]$. The global split is processed by a wavelet-enhanced state-space module. Flood extent, connectivity, and large-scale shape are represented mainly by smooth low-frequency structure, whereas water–land boundaries, narrow tributaries, and fragmented water bodies produce stronger directional high-frequency responses. Sending these components through a single undifferentiated long-range scan would couple regional semantic propagation with local boundary preservation. Accordingly, a discrete Haar wavelet transform decomposes $X_{g}$ into $X_{\mathrm{LL}}$, $X_{\mathrm{LH}}$, $X_{\mathrm{HL}}$, and $X_{\mathrm{HH}}$. SS2D models the low-frequency sub-band $X_{\mathrm{LL}}$ to capture long-range regional continuity, while the horizontal, vertical, and diagonal high-frequency sub-bands are preserved and refined locally before reconstruction. This design does not assume that Mamba cannot process high-frequency information. Rather, it assigns region-scale continuity to long-range state propagation while preserving boundary responses along a local path.

**Figure 3:**
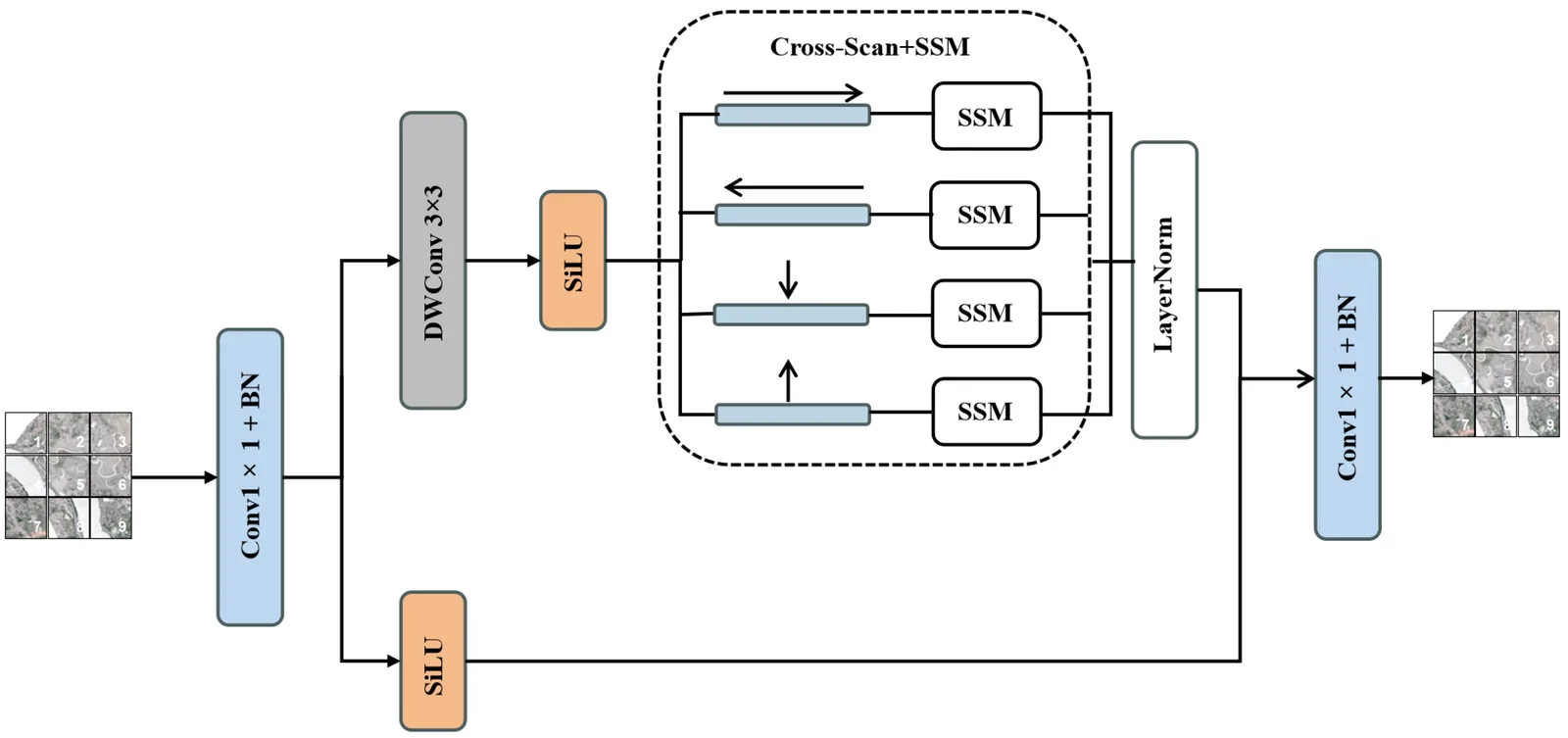
Forward data flow through the SS2D module with input-conditioned directional weighting. Numbers 1–9 label positions in the schematic 3×3 grid, and arrows within the Cross-Scan+SSM block indicate the four scan directions.

Figure 3 shows the input-dependent directional weighting used by the improved two-dimensional selective scan (SS2D) mechanism on $X_{\mathrm{LL}}$. The original SS2D reverses and sums the four directional scan outputs with equal weights, assigning the same directional importance regardless of the scene. The proposed lightweight direction gate instead learns per-sample weights, enabling the backbone to adapt its global directional preference to the current scene. The gate first applies global average pooling GAP(·) to $X_{\mathrm{LL}}$, then projects the result with $W_{d}$ and applies softmax to obtain the per-sample weights $w\in R^{B\times 4}$:

(3) $$w=\mathrm{Softmax}\left(W_{d}\cdot \mathrm{GAP}\left(X_{\mathrm{LL}}\right)\right)\in R^{B\times 4}$$

Each directional scan output is multiplied by its corresponding weight $w_{k}$ prior to merging. This enables the model to emphasize scan directions that are most relevant to the current scene, such as left-right scans for elongated flood corridors or top-bottom scans for vertical edges. The approach introduces only a single pooling-linear layer and preserves the selective scan kernel, resulting in the content-adaptive representation *Y*:

(4) $$Y=\sum_{k=1}^{4}w_{k}\cdot {\mathrm{Scan}}_{k}^{-1}\left(\mathrm{SSM}\left({\mathrm{Scan}}_{k}\left(X_{\mathrm{LL}}\right)\right)\right)$$

where $w_{k}$ is the learned weight of the *k*-th direction; ${\mathrm{Scan}}_{k}\left(\cdot \right)$ flattens the 2D feature map into a 1D token sequence along the *k*-th direction; ${\mathrm{Scan}}_{k}^{-1}\left(\cdot \right)$ restores the 2D layout; SSM(·) denotes the selective state-space scan; and *Y* is the resulting content-adaptive low-frequency representation.

The modeled low-frequency representation *Y* and the depthwise-refined high-frequency components ($X_{\mathrm{LH}},X_{\mathrm{HL}},X_{\mathrm{HH}}$) are subsequently reconstructed using an inverse discrete wavelet transform (IDWT) to produce ${\hat{X}}_{g}$:

(5) $${\hat{X}}_{g}=\mathrm{IDWT}\left(Y,\mathrm{DWConv}\left([X_{\mathrm{LH}}\|X_{\mathrm{HL}}\|X_{\mathrm{HH}}]\right)\right)+X_{g}$$

where IDWT(·) denotes the inverse discrete wavelet transform. The horizontal, vertical, and diagonal high-frequency sub-bands $X_{\mathrm{LH}}$, $X_{\mathrm{HL}}$, and $X_{\mathrm{HH}}$ encode structural boundary information. These components are refined by DWConv(·), while *Y* supplies the modeled global context. A residual connection combines the reconstructed global split ${\hat{X}}_{g}$ with the original split $X_{g}$.

Finally, ${\hat{X}}_{g}$ is concatenated with the depthwise-separable-convolution output of the local split and with the identity mapping. The concatenated features are then projected back to the original channel dimension to obtain the mixed representation:

(6) $$\mathrm{Mixer}\left(X^{\prime }\right)={\mathrm{Conv}}_{1\times 1}\left(\mathrm{ReLU}\left([{\hat{X}}_{g}\|\mathrm{DWSConv}\left(X_{l}\right)\|X_{\mathrm{id}}]\right)\right)$$

Two residual paths are retained in the block: one operates on the intermediate feature $X^{\prime }$, and the other links the mixed output to the input *X*. Regularization is supplied by DropPath. with the redundant operations after mixing removed from the original MobileMamba block, the output is

(7) $$X_{\mathrm{out}}=X+\mathrm{DropPath}\left(\mathrm{Mixer}\left(X^{\prime }\right)+X^{\prime }\right)$$

### 2.4. Deformable Scan Router Module

The third backbone limitation identified in Section 2.3 is the fixed geometry of standard two-dimensional selective scan (SS2D). Its rectilinear paths cannot conform to curved or fragmented flood boundaries. A straight scan may pass through inundated land, permanent water, and vegetation in sequence, causing the hidden state to mix semantically incompatible regions. The Deformable Scan Router (DSR) addresses this limitation directly. Inspired by deformable visual state space models [39], DSR retains the efficient scan operator while learning a content-adaptive spatial warp that makes the input sequence more responsive to boundary information.

The directional weighting described in Section 2.3 and DSR operate at different spatial levels. The sample-level direction weights identify which of the four global scan directions are more important, but they cannot alter the local traversal order around a curved or fragmented boundary. DSR provides this local flexibility by deforming the spatial layout at each position. Thus, directional weighting selects the global orientation, whereas DSR adapts the local path.

**Figure 4:**
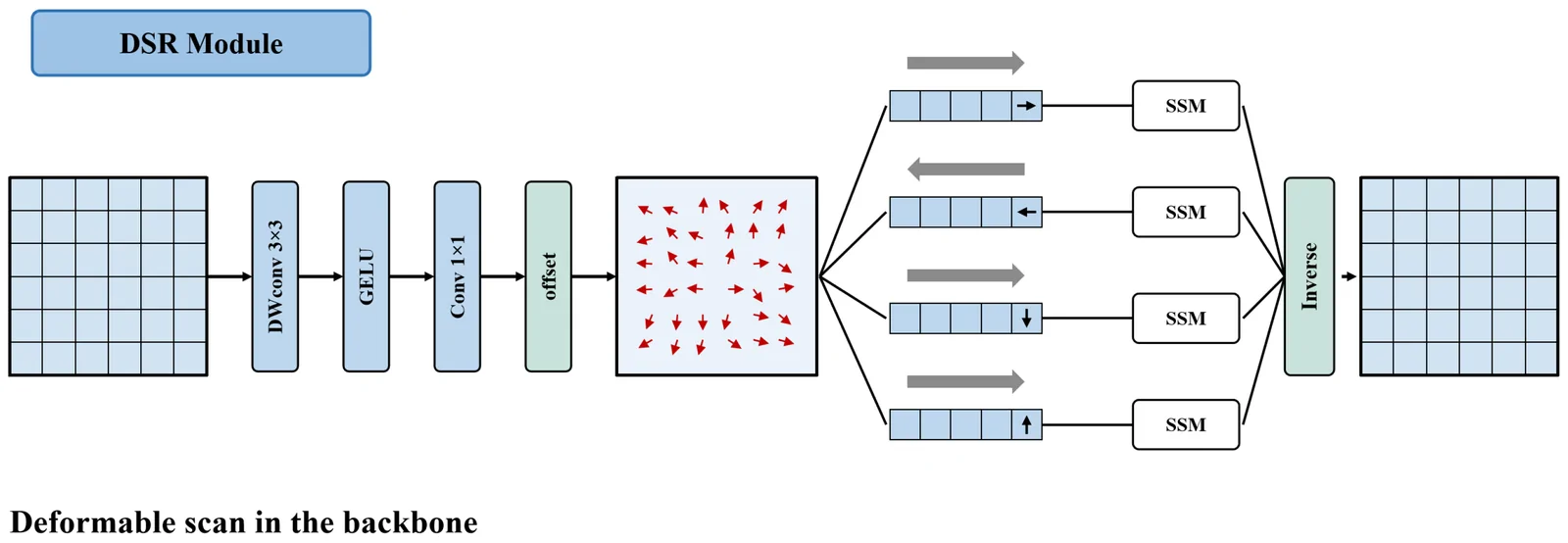
Deformable Scan Router (DSR). Per-token offsets redirect the scan from fixed rectilinear paths toward object boundaries. Red vectors denote learned spatial offsets, and gray arrows denote the four scan directions.

Direct modification of the state space model (SSM) scan indices would reduce computational efficiency and complicate backpropagation. DSR operates on the feature layout instead. The procedure in Figure 4 first warps the feature map, scans it in the standard order, and finally restores the original layout. For $X\in R^{B\times C\times H\times W}$, DSR constructs a normalized base grid $G_{\mathrm{base}}\in {\left[-1,1\right]}^{H\times W\times 2}$. Prediction is handled by a lightweight router $F_{\mathrm{router}}$; its output is the offset field ΔG:

(8) $$\Delta G=\alpha_{\mathrm{off}}F_{\mathrm{router}}\left(X\right),\;\alpha_{\mathrm{off}}=0.1$$

where ΔG contains the coordinate displacement assigned to every token. The coefficient $\alpha_{\mathrm{off}}=0.1$ scales ΔG in the normalized coordinate system. Because the output of $F_{\mathrm{router}}$ is not passed through a bounded activation, no explicit upper bound is imposed on the displacement magnitude before sampling.

Adding the offsets to the base grid and clamping the coordinates produces $G_{\mathrm{fwd}}=\mathrm{clamp}\left(G_{\mathrm{base}}+\Delta G,-1,1\right)$. Clipping therefore keeps all sampling locations within the valid grid domain. The bilinear sampler S(·,·) then uses this forward grid to reorganize the original features into a content-adaptive layout that follows the object boundaries:

(9) $$X_{w}=S\left(X,G_{\mathrm{fwd}}\right)$$

The warped feature map is denoted by $X_{w}$. Although the core SSM operator $\Phi_{\mathrm{ssm}}\left(\cdot \right)$ retains its fixed scan order, the preceding rearrangement makes the input sequence correspond more closely to the local boundary structure:

(10) $$Y_{w}=\Phi_{\mathrm{ssm}}\left(X_{w}\right)$$

Here, $Y_{w}$ represents the scan output in the warped layout. An inverse warp then restores spatial correspondence before FCM and RFI process the features. Under a first-order approximation, the inverse grid is $G_{\mathrm{inv}}=\mathrm{clamp}\left(G_{\mathrm{base}}-\Delta G,-1,1\right)$, and the restored feature is $Y=S(Y_{w},G_{\mathrm{inv}})$. The final offset projection is initialized to zero, so ΔG=0 and DSR is an identity mapping at the start of training. Consequently, the initial behavior is that of the original SSM, after which boundary-aware deformation is learned progressively. FCM and RFI then address a separate limitation outside the scope of the Mamba backbone. FCM controls region-dependent optical–SAR exchange, whereas RFI integrates the two branches while retaining modality differences.

### 2.5. Feature Complementary Module

Because optical–SAR relationships vary across a flood scene, FCM formulates cross-modal interaction as relationship-gated heterogeneous operator routing. A three-way gate generates local response maps associated with discrepancy, complementation, and consistency. Instead of selecting among interchangeable experts, these maps modulate three distinct operations: suppression of unreliable responses, bidirectional cross-attention for complementary information, and direct enhancement of mutually consistent evidence. The relationship-specific responses thereby govern cross-modal interaction at each location.

**Figure 5:**
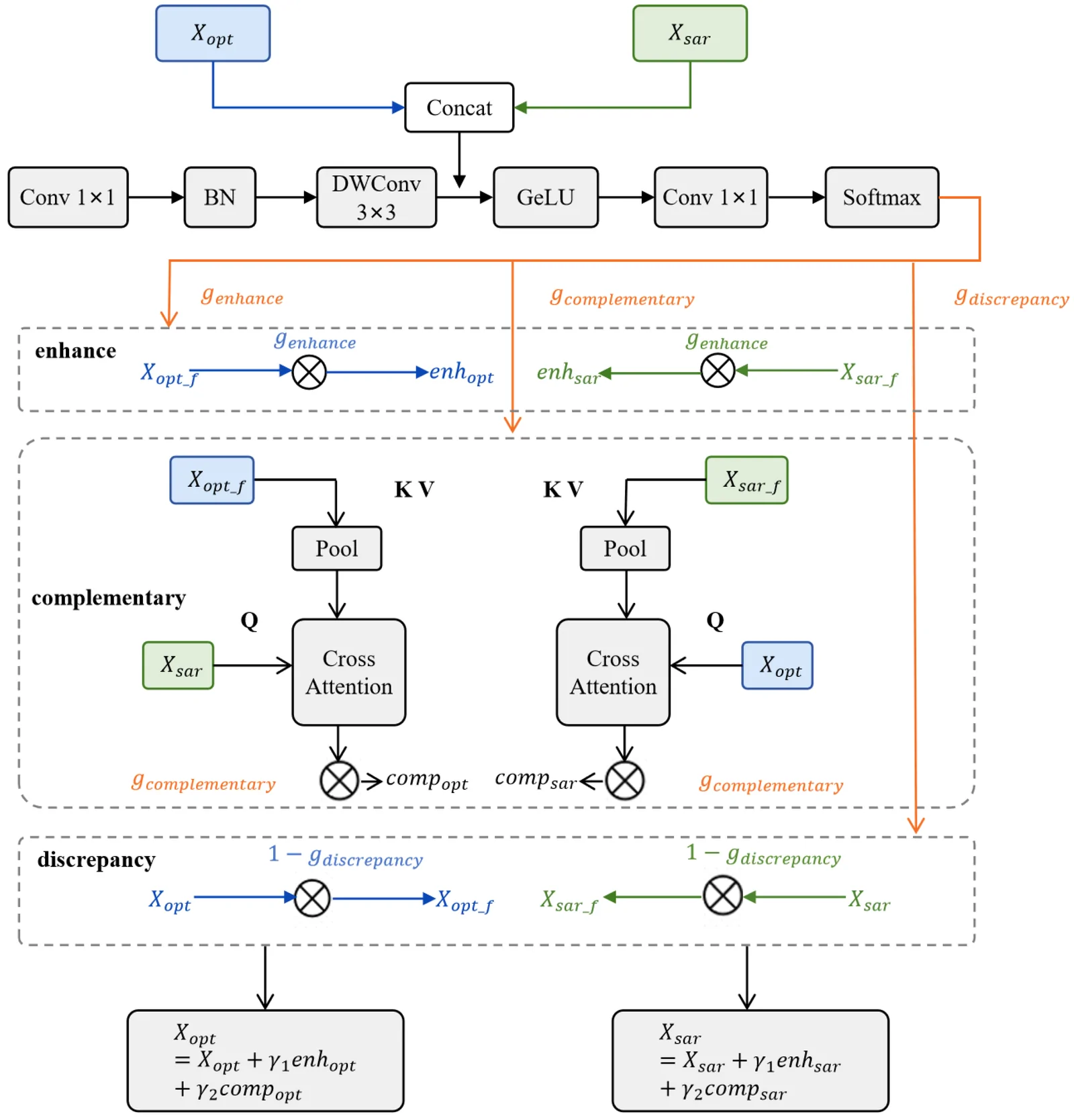
Structure of the Feature Complementary Module (FCM). Blue and green arrows denote the optical and SAR feature streams, respectively, and orange arrows denote the three relation gates.

As illustrated in Figure 5, FCM receives the optical feature $X_{\mathrm{opt}}$ and the SAR feature $X_{\mathrm{sar}}$. Concatenating them along the channel dimension gives $X_{\mathrm{cat}}\in R^{B\times 2C\times H\times W}$. The gating network first applies a 1×1 convolution, which reduces the channel dimension while allowing information from the two modalities to interact. A following 3×3 depthwise separable convolution captures local conditions, including cloud occlusion and speckle noise. The gate is computed as follows:

(11) $$G=\mathrm{Softmax}\left({\mathrm{Conv}}_{1\times 1}\left(\mathrm{GELU}({\mathrm{DWConv}}_{3\times 3}\left(\mathrm{BN}\left({\mathrm{Conv}}_{1\times 1}\left(X_{\mathrm{cat}}\right)\right)\right))\right)\right)$$

where softmax is applied across three channels at every position to produce $G\in R^{B\times 3\times H\times W}$. The tensor has three components: $G_{\mathrm{enh}}$ for enhancement, $G_{\mathrm{comp}}$ for complementation, and $G_{\mathrm{disc}}$ for discrepancy. Their sum is one at each spatial location, and the routing stage uses them as probabilities.

The routing probabilities determine how FCM treats each feature according to its local role. At locations dominated by $G_{\mathrm{disc}}$, the corresponding response is considered unreliable. FCM suppresses this response by retaining only the enhancement and complementary weights. For the optical branch, the resulting filtered feature is defined as follows:

(12) $$X_{\mathrm{opt}}^{f}=X_{\mathrm{opt}}⊙\left(G_{\mathrm{enh}}+G_{\mathrm{comp}}\right)$$

where ⊙ denotes element-wise multiplication. Applying the same operation to the other branch gives $X_{\mathrm{sar}}^{f}$.

For complementary regions, including cloud-contaminated optical areas where SAR remains informative, FCM uses bidirectional cross-attention with spatial reduction. The filtered SAR features are pooled before they are projected into the keys $K_{\mathrm{sar}}$ and values $V_{\mathrm{sar}}$, which lowers the cost relative to full cross-attention. At each scale, FCM operates on 64-channel embeddings with four attention heads ($d_{h}=16$) and applies a spatial-reduction ratio of 4 to the keys and values. Using the optical query $Q_{\mathrm{opt}}$ to attend to these pooled keys and values yields the restoration context $U_{\mathrm{opt}}$:

(13) $$U_{\mathrm{opt}}=\mathrm{Softmax}\left(\frac{Q_{\mathrm{opt}}K_{\mathrm{sar}}^{⊤}}{\sqrt{d_{h}}}\right)V_{\mathrm{sar}}⊙G_{\mathrm{comp}}$$

The optical features are projected into $Q_{\mathrm{opt}}$, whereas $K_{\mathrm{sar}}$ and $V_{\mathrm{sar}}$ come from the pooled SAR features. In the notation, ${\left(\cdot \right)}^{⊤}$ is the matrix transpose and $d_{h}$ is the per-head channel dimension that scales dot-product attention. The mask $G_{\mathrm{comp}}$ limits this response to complementary regions.

In aligned regions with a dominant $G_{\mathrm{enh}}$ response, the filtered counterpart from the other modality acts as a local enhancement signal:

(14) $$E_{\mathrm{opt}}=X_{\mathrm{sar}}^{f}⊙G_{\mathrm{enh}}$$

Because optical and SAR features have different noise characteristics, their learnable scales are kept separate in FCM. For the optical branch, the complementary context is weighted by $\gamma_{\mathrm{comp}}^{\mathrm{opt}}$ and the enhancement signal by $\gamma_{\mathrm{enh}}^{\mathrm{opt}}$. These terms combine to form $Y_{\mathrm{opt}}$, and an analogous expression is used for the SAR branch:

(15) $$Y_{\mathrm{opt}}=X_{\mathrm{opt}}+\gamma_{\mathrm{comp}}^{\mathrm{opt}}\cdot U_{\mathrm{opt}}+\gamma_{\mathrm{enh}}^{\mathrm{opt}}\cdot E_{\mathrm{opt}}$$

The four modality-specific scales $\gamma_{\mathrm{comp}}^{\mathrm{opt}}$, $\gamma_{\mathrm{comp}}^{\mathrm{sar}}$, $\gamma_{\mathrm{enh}}^{\mathrm{opt}}$, and $\gamma_{\mathrm{enh}}^{\mathrm{sar}}$ are each initialized to 0.1. Relative to the identity term $X_{\mathrm{opt}}$, they control the complementary and enhancement residuals. The modality-specific structure is therefore retained without preventing cross-modal compensation.

### 2.6. Refined Feature Integration

**Figure 6:**
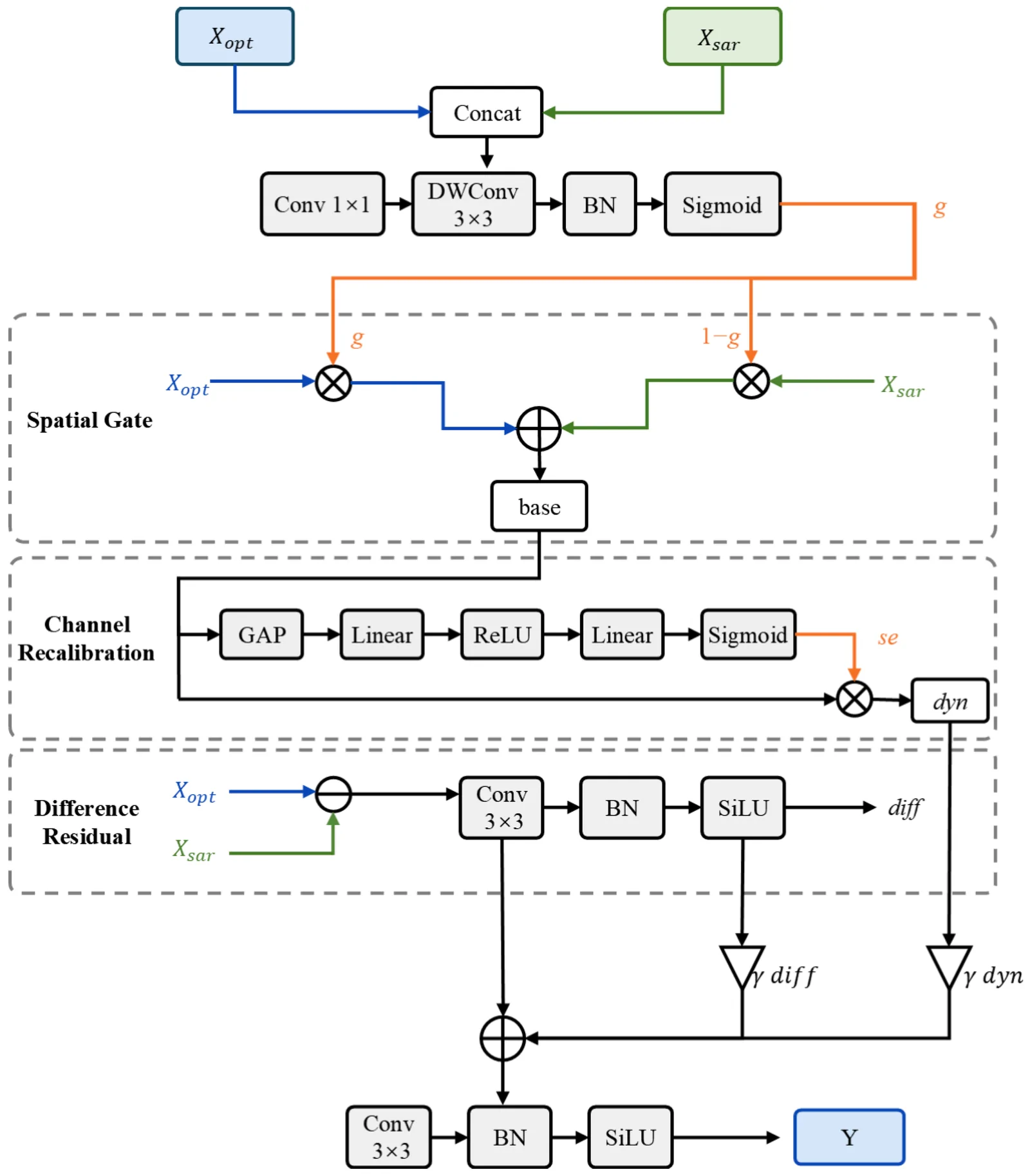
Structure of the Refined Feature Integration (RFI). Blue and green arrows denote the optical and SAR feature streams, respectively, and orange arrows denote learned gating weights.

FCM produces two relation-refined streams, which RFI combines through three-path disagreement-preserving residual integration. The first path forms a spatially gated base feature, the second recalibrates this shared response across channels, and the third projects the signed optical–SAR difference into an independent residual. Keeping the disagreement path separate from the shared representation preserves modality-specific evidence after fusion. As illustrated in Figure 6, the spatial gate is generated from the concatenated optical and SAR features by a 1×1 convolution followed by a 3×3 depthwise separable convolution:

(16) $$G=\sigma \left(\mathrm{BN}\left({\mathrm{DWConv}}_{3\times 3}\left({\mathrm{Conv}}_{1\times 1}\left(\left[X_{\mathrm{opt}},X_{\mathrm{sar}}\right]\right)\right)\right)\right)$$

where σ(·) denotes the sigmoid function, ensuring that $G\in {\left[0,1\right]}^{B\times C\times H\times W}$ functions as a per-pixel soft selector between the two modalities.

Subsequently, RFI applies channel-wise recalibration to the base fused feature using global average pooling (GAP) and fully connected layers:

(17) $$\begin{array}{cc} Y_{\mathrm{base}} & =G⊙X_{\mathrm{opt}}+\left(1-G\right)⊙X_{\mathrm{sar}}\\ Y_{\mathrm{dyn}} & =Y_{\mathrm{base}}⊙\sigma \left(F_{\mathrm{se}}\left(\mathrm{GAP}(Y_{\mathrm{base}})\right)\right) \end{array}$$

where $F_{\mathrm{se}}\left(\cdot \right)$ represents the squeeze-and-excitation sub-network operating on the pooled channel descriptor. $Y_{\mathrm{base}}$ and $Y_{\mathrm{dyn}}$ correspond to the spatially gated and channel-recalibrated features, respectively. The channel descriptor is reduced from 64 to 16 dimensions, giving a reduction ratio of 4.

Spatial-channel fusion alone remains susceptible to modality collapse. Due to differing statistical properties and noise patterns, the optimization rates of optical and SAR features are not always aligned. During training, the network may prioritize the branch that is more readily optimized; for example, clear optical textures can suppress SAR contributions, whereas significant cloud cover may bias the model toward SAR. Both scenarios compromise the intended robustness of the dual-stream architecture.

The third path explicitly retains the signed disagreement between the two sensors instead of relying solely on the convex combination above. The differential feature $Y_{\mathrm{diff}}$ is computed using a projection layer $F_{\mathrm{diff}}$:

(18) $$Y_{\mathrm{diff}}=F_{\mathrm{diff}}\left(X_{\mathrm{opt}}-X_{\mathrm{sar}}\right)$$

During final aggregation, the differential feature is combined with the channel-recalibrated feature. This residual path keeps disagreement between the optical and SAR features visible throughout fusion:

(19) $$Y_{\mathrm{out}}=F_{\mathrm{out}}\left(Y_{\mathrm{base}}+\gamma_{\mathrm{dyn}}\cdot Y_{\mathrm{dyn}}+\gamma_{\mathrm{diff}}\cdot Y_{\mathrm{diff}}\right)$$

where $Y_{\mathrm{out}}$ is the fused representation, and $F_{\mathrm{diff}}$ and $F_{\mathrm{out}}$ are convolutional blocks. A 3×3 convolution, batch normalization, and a SiLU activation in $F_{\mathrm{out}}$ smooth the residual mixture and map it to the decoder space. Both $\gamma_{\mathrm{dyn}}$ and $\gamma_{\mathrm{diff}}$ are initialized to 0.1. This choice allows dynamic recalibration and the difference residual to influence the representation from the beginning of training. Through these two paths, RFI balances optical–SAR integration while preserving boundary continuity under degraded observations.

### 2.7. Spectral Refinement Block

**Figure 7:**
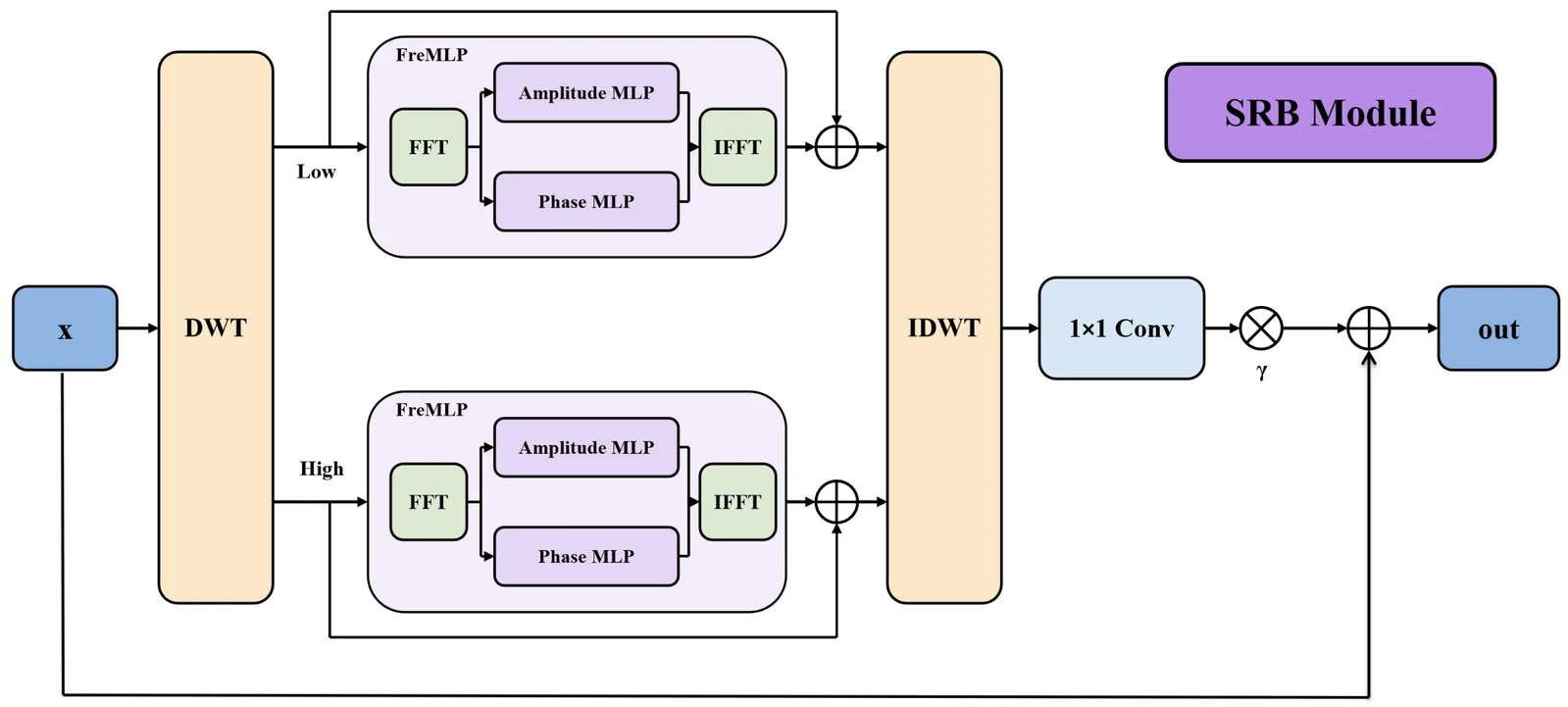
The Spectral Refinement Block (SRB), which decomposes features by a discrete wavelet transform and refines the low- and high-frequency sub-bands with frequency-domain MLPs to recover high-frequency boundary details.

Deep fusion and repeated downsampling tend to weaken the high-frequency information carried by object boundaries. The Spectral Refinement Block (SRB) restores this information at the decoder output as shown in Figure 7. Here, the discrete wavelet transform (DWT) is preferred to the Fourier transform because it separates frequency components while retaining their spatial locations. Following recent work on wavelet-domain modeling [40], the input feature *X* is decomposed as follows:

(20) $$\mathrm{DWT}\left(X\right)=\left\{X_{\mathrm{LL}},X_{\mathrm{LH}},X_{\mathrm{HL}},X_{\mathrm{HH}}\right\}$$

where $X_{\mathrm{LL}}$ is the low-frequency approximation of the structure. The other three terms, $X_{\mathrm{LH}}$, $X_{\mathrm{HL}}$, and $X_{\mathrm{HH}}$, contain the horizontal, vertical, and diagonal high-frequency components, respectively.

SRB keeps $X_{\mathrm{LL}}$ as its low-frequency approximation and concatenates the remaining three sub-bands to form the high-frequency representation $X_{\mathrm{high}}$:

(21) $$X_{\mathrm{high}}=\mathrm{Concat}\left(\left[X_{\mathrm{LH}},X_{\mathrm{HL}},X_{\mathrm{HH}}\right]\right)$$

Combining the sub-bands in this way removes the need to maintain three separate high-frequency branches. Because each sub-band represents gradients in a different direction, the layers that follow can learn how horizontal, vertical, and diagonal responses relate to one another. Such relationships are particularly relevant to flood contours, which may include curved segments, corners, and fragmented edges.

Once the decomposition is complete, SRB carries out refinement in two streams. The learnable frequency MLPs $\Psi_{L}$ and $\Psi_{H}$ refine the low- and high-frequency representations, respectively, retaining coarse structure while enhancing boundary responses. A zero-initialized residual connection reconstructs the refined components and returns them to the main stream:

(22) $$Y=X+\gamma ⊙F_{\mathrm{proj}}\left(W^{-1}\left(\Psi_{L}\left(X_{\mathrm{LL}}\right),\;\Psi_{H}\left(X_{\mathrm{high}}\right)\right)\right)$$

Here, $W^{-1}\left(\cdot \right)$ denotes the inverse discrete wavelet transform, $\Psi_{L}$ and $\Psi_{H}$ are learnable frequency MLPs, and $F_{\mathrm{proj}}$ is a 1×1 convolution for channel alignment. The channel-wise scale γ contains 64 coefficients, all initialized to zero. Consequently, SRB begins as an identity mapping and gradually learns spectral refinement.

### 2.8. Loss Function

Flood scenes are highly imbalanced because inundated foreground pixels are much less numerous than background pixels. The training objective therefore combines a Binary Cross-Entropy (BCE) term with a Dice term. The BCE component provides stable gradients at the pixel level, whereas the Dice component acts directly on regional overlap and encourages sharper boundary delineation:

(23) $$L_{\mathrm{bce}}=-\frac{1}{N}\sum_{i=1}^{N}\left[g_{i}\log\left(p_{i}\right)+\left(1-g_{i}\right)\log\left(1-p_{i}\right)\right]$$

(24) $$L_{\mathrm{dice}}=1-\frac{2\sum_{i=1}^{N}p_{i}g_{i}+\epsilon }{\sum_{i=1}^{N}p_{i}+\sum_{i=1}^{N}g_{i}+\epsilon }$$

The two loss terms are combined linearly so that pixel-level accuracy is balanced with coherence over the entire predicted region:

(25) $$L_{\mathrm{total}}=\lambda_{1}L_{\mathrm{bce}}+\lambda_{2}L_{\mathrm{dice}}$$

where *N* is the number of pixels, $p_{i}\in \left[0,1\right]$ gives the predicted probability for pixel *i*, and $g_{i}\in \left\{0,1\right\}$ is the corresponding ground-truth label. The small constant $\epsilon =10^{-5}$ stabilizes the numerical computation. The weights $\lambda_{1}$ and $\lambda_{2}$ are set empirically to $\lambda_{1}=\lambda_{2}=0.5$, so the BCE and Dice terms contribute equally to the objective.

## 3. Experimental Results

### 3.1. Datasets

#### 3.1.1. CAU-Flood

CAU-Flood [25] is a large-scale optical–SAR benchmark developed for flood-inundation mapping. Its main change-detection protocol pairs pre-disaster Sentinel-2 optical imagery with post-disaster Sentinel-1 SAR observations. The optical input comprises Sentinel-2 bands B2 (blue), B3 (green), B4 (red), and B8 (near-infrared). The SAR observations are derived from Sentinel-1 Ground Range Detected (GRD) products. The dataset provider processes only the VV polarization, producing one grayscale backscatter channel for each SAR patch. Where cloud-free pre-disaster optical imagery was unavailable, post-disaster Sentinel-2 imagery was used instead. We use the released pairings and their flood-label semantics as provided, without imposing an additional same-day or fixed-interval requirement. The 18 study plots span $95,142\;{\mathrm{km}}^{2}$ across China, Bangladesh, Australia, the United States, Canada, and Germany, covering river systems, lacustrine basins, and urban landscapes.

CAU-Flood contains 18,302 co-registered patches, each measuring 256×256 pixels. The training and test partitions contain 15,231 and 3071 patches, respectively. During dataset construction, the Sentinel-2 and Sentinel-1 products were first referenced to WGS84. Residual cross-sensor offsets were then corrected manually in ArcGIS [25] using at least nine ground control points selected from stable structures. Each pair was subsequently resampled to the same image size before patch extraction. A refined filter reduces SAR speckle, while digital elevation models (DEMs) at 30 m spatial resolution mask regions whose slopes exceed 5° to limit interference from terrain shadows. All of these operations—SAR filtering, terrain correction, cross-sensor registration, slope-based terrain masking, resampling, and patch construction—were performed by the CAU-Flood developers during benchmark preparation. We use the complete released dataset, including the optical images, VV-polarized SAR images, labels, and official training/test split, without applying any additional remote-sensing preprocessing. Nevertheless, environmental noise, temporal land-cover changes, and residual misregistration may still produce cross-modal discrepancies.

#### 3.1.2. Wuhan

The Wuhan dataset [42] is a regional benchmark built around the severe waterlogging and urban flooding that affected Wuhan, Hubei Province, China, in July 2016. The dataset pairs Sentinel-2 optical imagery with a single-channel amplitude image derived from an HH-polarized COSMO-SkyMed acquisition in StripMap HIMAGE mode. This amplitude image serves as the SAR input. In contrast to the larger CAU-Flood benchmark, Wuhan contains dense urban textures in which floodwater is interspersed with buildings, roads, and vegetation, making the boundaries difficult to delineate. The optical observations are also degraded by weather interference; SAR evidence is therefore especially important during fusion. The dataset comprises 664 high-quality image pairs, with each image measuring 128×125 pixels. Following previous studies, 552 pairs are assigned to training and 112 to testing, which creates a data-scarce evaluation setting.

#### 3.1.3. Analysis

The evaluation assigns a different role to each dataset. Because CAU-Flood is larger and covers more heterogeneous scenes, it is used for the ablation studies and hyperparameter analysis. Its scale provides a more stable basis for estimating how FCM, RFI, DSR, and SRB contribute to the final result.

Wuhan, in comparison, provides the more difficult data-scarce case. The 664 image pairs and the dense urban structure represented in the dataset make it possible to examine performance with few training examples and degraded optical observations.

#### 3.1.4. Temporal and Spatial Input Requirements

Spatially, each optical–SAR pair supplied to CISRMamba must cover the same geographic extent and be geometrically registered to a common spatial reference and sampling grid. Although the sensors may have different native spatial resolutions, their input products must be resampled onto the same H×W grid so that corresponding pixels refer to the same geographic locations. Merely resizing two unregistered arrays to identical dimensions does not establish this correspondence, and CISRMamba does not perform image registration internally.

Temporally, the optical and SAR observations need not be acquired simultaneously. The architecture uses neither acquisition timestamps nor inter-image intervals as inputs and does not impose a fixed temporal gap. However, the event relationship and label semantics of each pair must remain consistent with the training protocol. CAU-Flood primarily uses pre-disaster Sentinel-2 optical imagery and post-disaster Sentinel-1 SAR observations, with documented substitutions of post-disaster optical imagery where cloud-free pre-disaster observations were unavailable. The present experiments evaluate only the released CAU-Flood and Wuhan pairings; they do not establish equal performance for arbitrary temporal gaps, reversed acquisition order, or residual spatial misregistration.

### 3.2. Experimental Setup

#### 3.2.1. Baselines

The comparison covers ten representative methods from the CNN, Transformer, and Mamba families. Established and task-specific CNN baselines include UNet++ [43], DeepLabV3+ [44], CMCDNet [25], CCDCNet [45], HANet [46], and RFANet [47]. SemTFormer [48] and UCDFormer [49] form the Transformer group, whereas MambaSeg [50] and SD-Mamba [37] are the recent Mamba baselines. For a consistent comparison, every baseline is retrained from its official implementation on the same datasets and with the same training setup.

#### 3.2.2. Implementation Details

All experiments are implemented in PyTorch 2.0.0 and run on a workstation with a single NVIDIA GeForce RTX 4090 GPU (NVIDIA Corporation, Santa Clara, CA, USA). Improved MobileMamba [41] serves as the feature-extraction backbone. No external pretraining is used, and all network weights are learned from scratch. Inputs are resized to 256×256 pixels, which requires the original 128×125 Wuhan images to be upsampled. This resizing follows the established Wuhan evaluation protocol: the original Wuhan study uses 256×256 network inputs, and the official SD-Mamba implementation likewise standardizes the datasets as 256×256 patches [37,42]. Because resampling can smooth high-frequency boundary detail, the Wuhan results should be interpreted on the resized grid rather than at the native 128×125 sampling. The same transformation is applied to every compared method and its corresponding labels, preserving a controlled relative comparison without favoring a particular architecture. For both datasets, the released grayscale SAR images are converted to floating-point values, scaled from 0–255 to 0–1, and supplied to the single-channel SAR encoder. No additional polarization channel or hand-crafted SAR feature is introduced. Training lasts 60 epochs with a batch size of 16. The AdamW optimizer uses a weight decay of 0.01, while a cosine schedule decreases the learning rate from $1\times 10^{-4}$ to $1\times 10^{-6}$. During training, horizontal and vertical flips are applied independently to each image pair with a probability of 0.5; validation and test samples are not augmented. Images are resized by bilinear interpolation, binary masks by nearest-neighbor interpolation, and all experiments use a random seed of 42.

### 3.3. Evaluation Metrics

Five primary segmentation metrics are used for evaluation: mean intersection over union (mIoU), overall accuracy (Acc), F1-score (F1), precision, and recall. For every test image, a pixel-level confusion matrix is first constructed with water designated as the positive class. The five metrics are calculated from this image-specific matrix, and the reported values are their arithmetic means across the test images. In the notation below, TP, FP, TN, and FN denote true positives, false positives, true negatives, and false negatives, respectively.

Precision measures the proportion of predicted water pixels that are classified correctly:

(26) $$\mathrm{Precision}=\frac{\mathrm{TP}}{\mathrm{TP}+\mathrm{FP}}$$

Recall measures the proportion of ground-truth water pixels detected by the model:

(27) $$\mathrm{Recall}=\frac{\mathrm{TP}}{\mathrm{TP}+\mathrm{FN}}$$

Because flood scenes usually have a severe foreground-background imbalance, the F1-score is computed as the harmonic mean of precision and recall, balancing the two quantities:

(28) $$F1=2\times \frac{\mathrm{Precision}\times \mathrm{Recall}}{\mathrm{Precision}+\mathrm{Recall}}$$

Overall accuracy is the proportion of all pixels assigned to the correct class:

(29) $$\mathrm{Acc}=\frac{\mathrm{TP}+\mathrm{TN}}{\mathrm{TP}+\mathrm{TN}+\mathrm{FP}+\mathrm{FN}}$$

Finally, mIoU is used as the primary segmentation metric. It averages the intersection-over-union over all *N* classes, where N=2 for binary water detection:

(30) $$\mathrm{mIoU}=\frac{1}{N}\sum_{i=1}^{N}\frac{{\mathrm{TP}}_{i}}{{\mathrm{TP}}_{i}+{\mathrm{FP}}_{i}+{\mathrm{FN}}_{i}}$$

Because flood mapping is a binary task, we also report the IoU for the positive water class:

(31) $${\mathrm{IoU}}_{\mathrm{water}}=\frac{\mathrm{TP}}{\mathrm{TP}+\mathrm{FP}+\mathrm{FN}}.$$

For the class-specific CAU-Flood evaluation, TP, FP, and FN are accumulated over the complete test set before calculating Water IoU.

Boundary quality is evaluated using boundary F1 scores at matching tolerances of 1, 2, and 3 pixels (BF@1, BF@2, and BF@3), together with Boundary IoU computed within a fixed 3-pixel boundary band. Predicted probability maps are binarized at a threshold of 0.5, and identical contour-extraction and boundary-matching settings are used for all variants.

### 3.4. Comparative Results

#### 3.4.1. Comparison of Model Performance on the CAU-Flood Dataset

The CAU-Flood results for CISRMamba and the ten baseline methods are reported in Table 1. CISRMamba ranks first on all five evaluation metrics.

Among the earlier convolutional neural network (CNN) baselines, HANet, RFANet, DeepLabV3+, and UNet++ obtain mean intersection over union (mIoU) values ranging from 85.41% to 88.33%. Cross-modal CNNs improve on this range, and CCDCNet reaches 89.85%. Nevertheless, these architectures still depend on local receptive fields, which restricts the global context available to the model.

Self-attention enables SemTFormer and UCDFormer to represent longer-range relationships, but this ability comes with considerable computational cost. MambaSeg and SD-Mamba take a different approach, using linear-complexity state space models (SSMs); both achieve more than 90% mIoU. However, the fixed scan patterns in these models remain less well matched to irregular flood boundaries.

To maintain consistent metric coverage across methods, Table 1 retains mIoU as the common IoU-based comparison metric. Water IoU is not included as a table column because it is unavailable for several baselines. Instead, we report a separate class-specific comparison with CCDCNet, CMCDNet, and SD-Mamba. We selected these three methods because they are strong baselines developed specifically for optical–SAR flood mapping [25,37,45]. Under the unified evaluation protocol on the complete CAU-Flood test set, CISRMamba achieves a Water IoU of 86.03%, exceeding CCDCNet (83.57%), CMCDNet (83.15%), and SD-Mamba (80.90%) by 2.46, 2.88, and 5.13 percentage points, respectively. These gains indicate that the water regions predicted by CISRMamba have greater spatial agreement with the reference regions.

For CAU-Flood, CISRMamba reaches 91.53% mean intersection over union (mIoU) and 95.47% F1-score. Compared with the second-ranked SD-Mamba, the respective margins are 1.26 and 0.72 percentage points. The model also gives the highest precision, 95.23%, and the highest recall, 95.73%, among all methods in the table. The gains thus extend across multiple metrics rather than a single score. The higher precision corresponds to fewer false alarms on difficult surfaces such as bare soil, buildings, and shadowed areas, while the higher recall indicates that fewer narrow or weak-response water regions are missed. This precision–recall balance suggests that FCM and RFI reduce modality bias during optical–SAR fusion, whereas DSR and SRB preserve boundary consistency and fine spatial detail.

**Figure 8:**
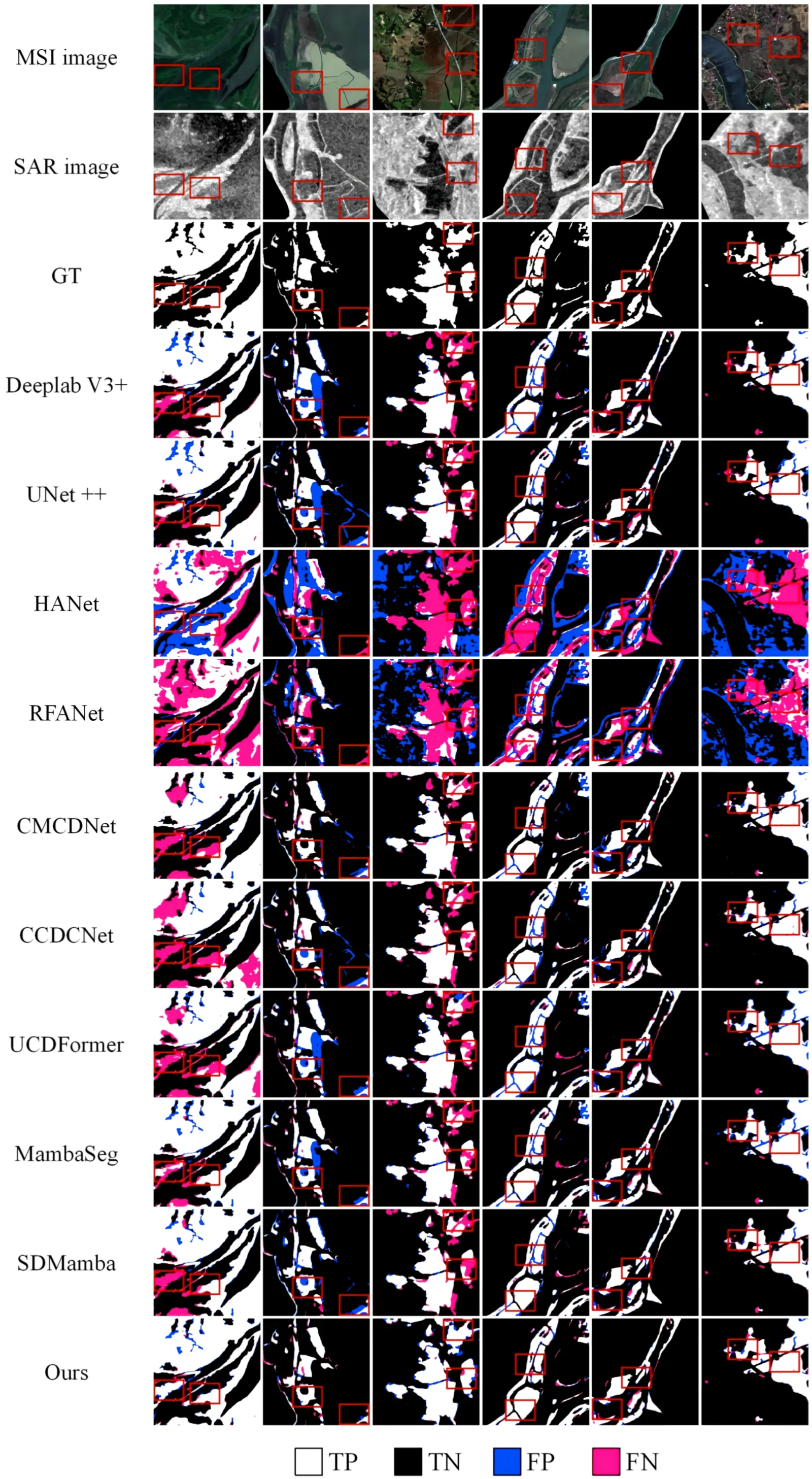
Visual comparison of flood-inundation maps on the CAU-Flood dataset. Red boxes mark regions where the prediction maps differ markedly across methods.

Figure 8 presents the predicted CAU-Flood maps alongside the MSI and SAR inputs. Columns 1 to 3 contain large, irregularly shaped water bodies against noisy terrain. In these cases, HANet and RFANet produce many false positives (blue), often labeling bare soil or buildings as water. CCDCNet and UCDFormer remove part of this interference; however, scattered responses remain over heterogeneous land cover. CISRMamba suppresses these false alarms more consistently. This behavior agrees with the intended role of FCM, which filters unreliable cross-modal evidence before feature integration.

The examples in columns 4 to 6 instead contain narrow tributaries and fragmented water patches. UNet++ and DeepLabV3+ frequently break these structures because local convolutional features do not connect thin water regions reliably. MambaSeg and SD-Mamba provide broader context, yet their fixed scan routes may still miss small water bodies or over-smooth curved edges. CISRMamba maintains the continuity of thin rivers more effectively and also retains isolated patches and irregular boundaries.

Closer inspection of the red boxes shows a fairly consistent difference. In the CISRMamba row, the blue and magenta errors are mostly narrow bands along the water boundary. Several competing predictions, by contrast, carry these errors farther into the flooded area or the surrounding land. The qualitative result cannot separate the contribution of each block, but it follows the trend in the ablation study: FCM and RFI limit errors associated with unreliable cross-modal responses, whereas DSR and SRB retain narrow structures and local boundary detail. The improvement is therefore visible in the spatial distribution of the errors, not only in the aggregate metrics.

#### 3.4.2. Comparison of Model Performance on the Wuhan Dataset

Table 2 reports the results of all models on Wuhan. The dataset provides only 552 training pairs and contains a complex urban layout, conditions that increase the risk of overfitting and modality collapse when the optical evidence is weak.

As Table 2 shows, CISRMamba records the highest mIoU (58.67%), accuracy (87.67%), F1-score (70.65%), and recall (68.56%). With the same limited amount of training data, most CNN and Transformer baselines remain below 56% mIoU. Wuhan combines dense buildings, fragmented water bodies, and weather-degraded optical observations, making fusion especially sensitive to imbalance between the modalities. Although the alternative Mamba models are lighter, their fixed scans still restrict performance near complex urban flood boundaries.

The precision–recall relationship further distinguishes the methods. UCDFormer has the highest precision at 73.40% but reaches only 62.59% recall. SD-Mamba shows a similar pattern, with 72.64% precision and 64.48% recall. On Wuhan, such conservative predictions miss more tributaries and leave flood boundaries blurred.

At 72.89%, the precision of CISRMamba remains competitive, while its recall reaches 68.56%. This recall is 5.97 percentage points higher than that of UCDFormer and 4.08 points higher than that of SD-Mamba. The model also ranks first in mean intersection over union (mIoU), F1-score, and accuracy. Thus, the gain does not simply reflect a precision–recall trade-off: more true flood pixels are recovered without losing control of false alarms. Consequently, CISRMamba is less conservative than the high-precision baselines, but it does not become overly permissive. The result is consistent with the roles of FCM and RFI, which incorporate local complementary cues while the difference residual prevents one modality from dominating.

The gap between CISRMamba and the second-best method is wider on Wuhan than on CAU-Flood. It leads UNet++ by 2.65 percentage points on Wuhan, compared with a 1.26-point lead over SD-Mamba on CAU-Flood. The ordering of the baselines changes across the two datasets, whereas CISRMamba remains first in both cases.

**Figure 9:**
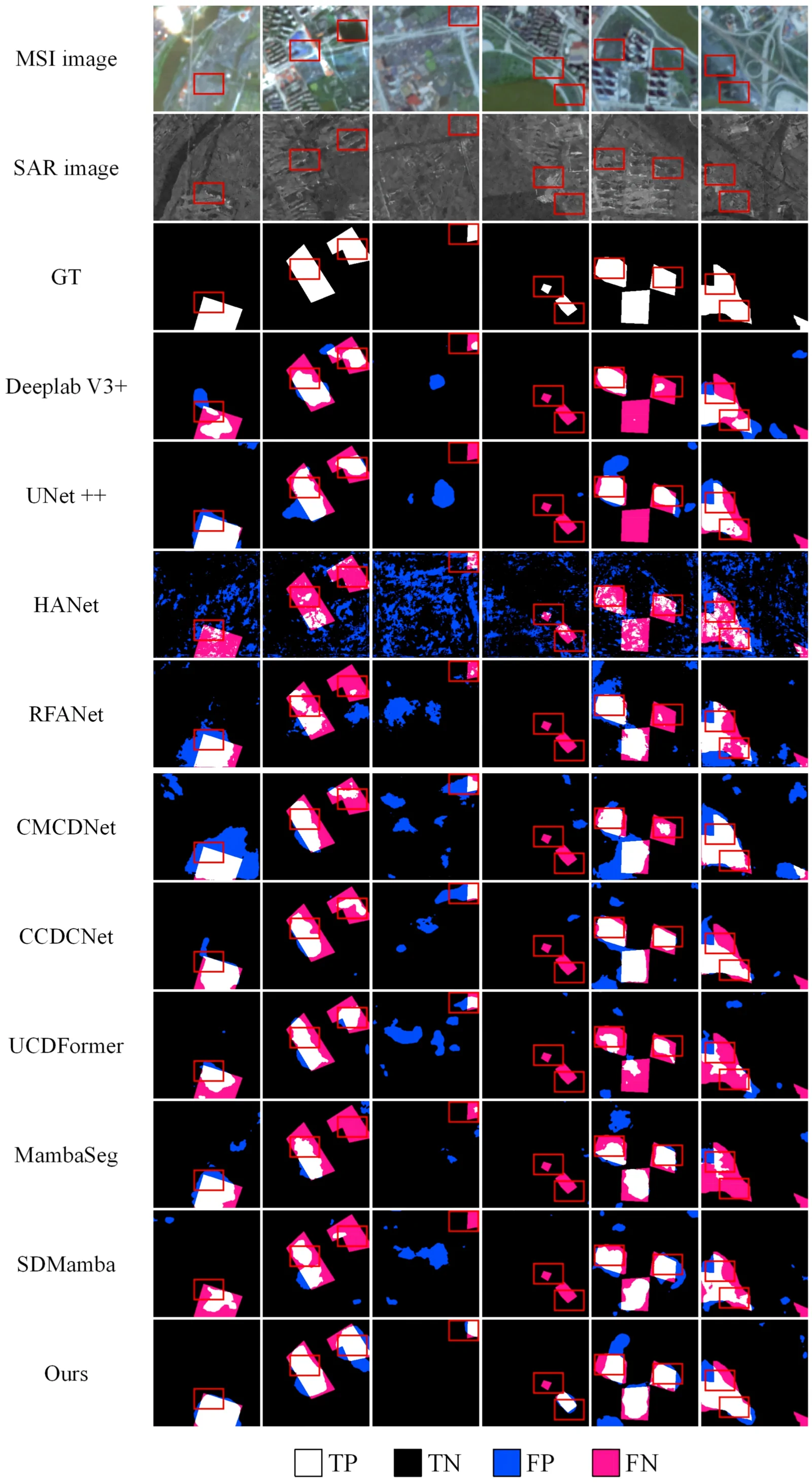
Visual comparison of flood-segmentation maps on the Wuhan dataset. Red boxes mark regions where the prediction maps differ markedly across methods.

Representative Wuhan results are shown in Figure 9. All methods perform worse than on CAU-Flood because the training set is smaller and the urban background is more complex. HANet produces fragmented maps and many false alarms, while the convolutional neural network (CNN) baselines tend to blur contours and omit small tributaries. UCDFormer, MambaSeg, and SD-Mamba recover the main water bodies, but still miss narrow or curved structures. For these difficult regions, CISRMamba produces predictions that more closely match the ground truth, particularly for small tributaries and water fragments between built-up areas. SAR supplies useful evidence in such locations, although improper fusion can allow optical texture to obscure it. The observed pattern agrees with Table 2 and supports region-dependent multi-sensor fusion rather than uniform alignment.

The Wuhan cases show a similar, although less clear-cut, pattern. CISRMamba still misses parts of some boundaries, but these regions are generally smaller and less scattered than those produced by most baselines. It also recovers more of the small flooded patches. The same tendency on Wuhan suggests that the modules remain useful when the local optical and SAR responses disagree.

## 4. Discussion

### 4.1. Ablation Study

**Quantitative component ablation.** The ablation experiments use CAU-Flood, whose larger scale limits the effect of small-sample variation on the module analysis. The baseline excludes FCM, RFI, and SRB. Table 3 reports the changes obtained when these modules are added individually or together. DSR remains enabled in these configurations. To evaluate it under matched conditions, Table 3 also includes a w/o-DSR configuration that retains FCM, RFI, and SRB.

With all three modules removed, the baseline reaches 89.51% mean intersection over union (mIoU) and a 92.99% F1-score. Adding any one of the modules improves the result. FCM produces a 1.01-percentage-point increase in mIoU, supporting region-dependent fusion rather than a fixed align-then-fuse operation. RFI contributes 0.88 points by retaining cross-modal differences during integration, which reduces modality collapse. SRB uses the discrete wavelet transform (DWT) to recover high-frequency boundary cues and increases mIoU by 0.47 points.

The results also show a complementary effect between FCM and RFI. When both are enabled, precision reaches 95.70%, which is the highest value reported in Table 3. This result indicates that context-aware routing and difference-guided integration jointly reduce false positives associated with optical–SAR distribution shifts.

With FCM, RFI, and SRB held constant, removing DSR reduces mIoU from 91.53% to 91.11%, a decrease of 0.42 percentage points. This controlled comparison isolates the contribution of DSR within the complete fusion architecture. With all four modules enabled, the full model achieves the highest mIoU (91.53%), F1-score (95.47%), and recall (95.73%). Compared with the baseline that retains DSR but excludes FCM, RFI, and SRB, the full configuration improves mIoU by 2.02 percentage points while adding 0.68 million parameters and 1.02 GMACs.

**Boundary-specific ablation.** Because region-overlap metrics do not directly characterize contour quality, Table 4 evaluates the separate and combined contributions of DSR and SRB with boundary-specific measures while holding the remainder of the architecture fixed.

Relative to the configuration without DSR and SRB, enabling DSR alone improves BF@1, BF@2, BF@3, and Boundary IoU by 2.09, 2.12, 1.27, and 2.77 percentage points, respectively; enabling SRB alone yields corresponding gains of 2.80, 1.62, 1.72, and 3.04 points. Their joint use produces the highest value for every boundary metric, increasing BF@1, BF@2, BF@3, and Boundary IoU by 4.49, 3.44, 2.87, and 4.05 points, respectively. The consistent gains at all three matching tolerances demonstrate the contribution of both modules to boundary quality, with the combined configuration providing the highest overall contour accuracy.

**Figure 10:**
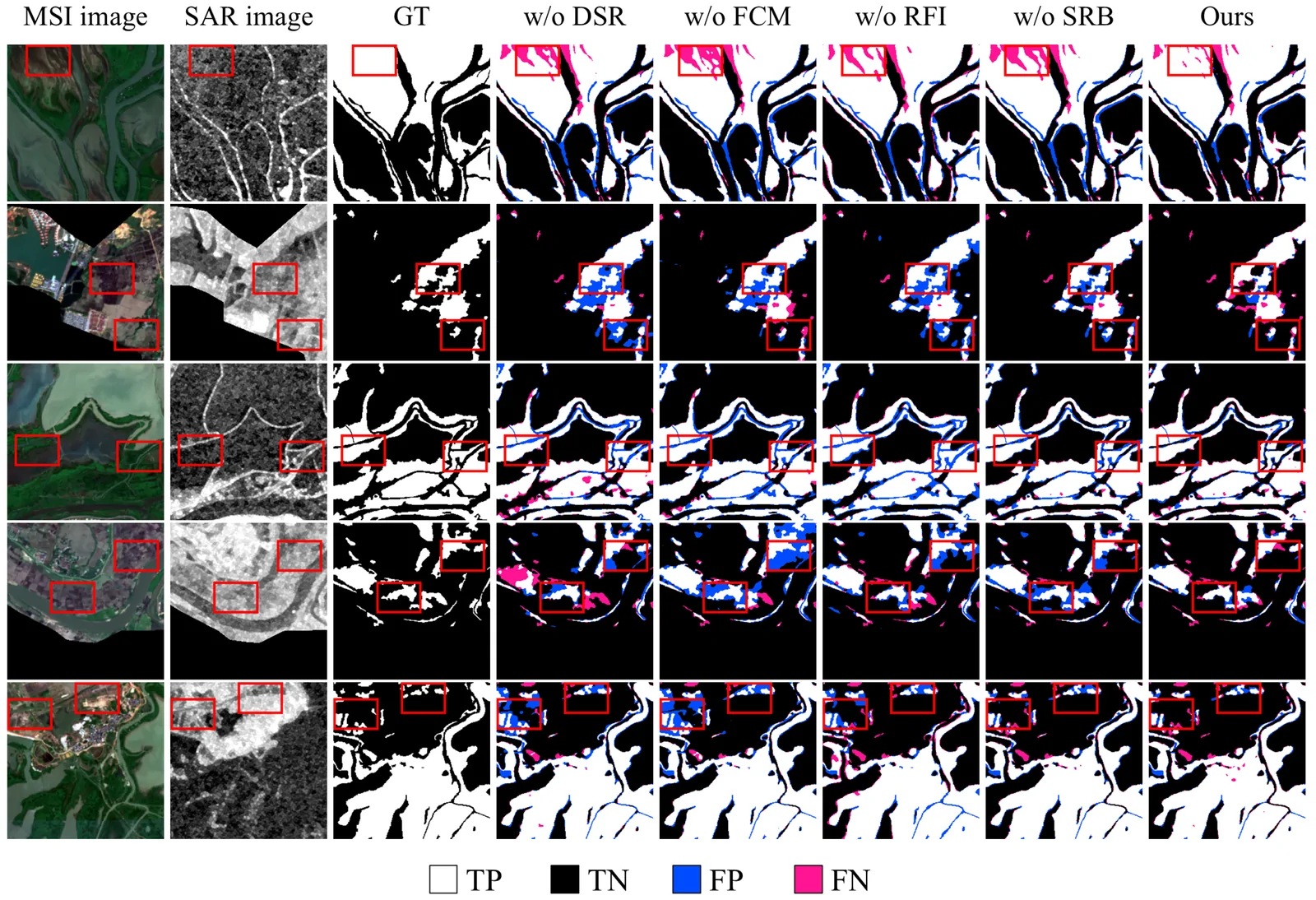
Visual comparison of component-ablation results on the CAU-Flood dataset. Red boxes mark regions where predictions differ markedly across model configurations.

**Qualitative component ablation.** Figure 10 compares the full model with variants obtained by removing one component at a time.

The five CAU-Flood cases span large, medium, and small flooded areas. Each row shows, from left to right, the MSI image, SAR image, ground truth (GT), results without DSR, FCM, RFI, or SRB, and the full-model result. White and black indicate true positives and true negatives, whereas blue and magenta indicate false positives and false negatives. The same red boxes mark the comparison regions within each row.

Most differences are concentrated around narrow channels, curved water–land boundaries, and isolated water patches. Removing a component introduces additional blue or magenta pixels in these regions, whereas the full-model prediction generally remains closer to the ground truth. This pattern is consistent with the modest gains in Table 3. The boundary-specific results in Table 4 provide the corresponding quantitative contour evaluation. Because the figure presents prediction outputs, it supports the contribution of the components but cannot independently establish the internal routing or scanning behavior of any individual module.

### 4.2. Empirical Analysis of Cross-Modal Information Utilization

Aggregate segmentation scores do not show how the network uses heterogeneous observations. We therefore first examine the spatial routing responses learned by FCM and then quantify the contribution of each modality and the effect of RFI under controlled input interventions.

**Figure 11:**
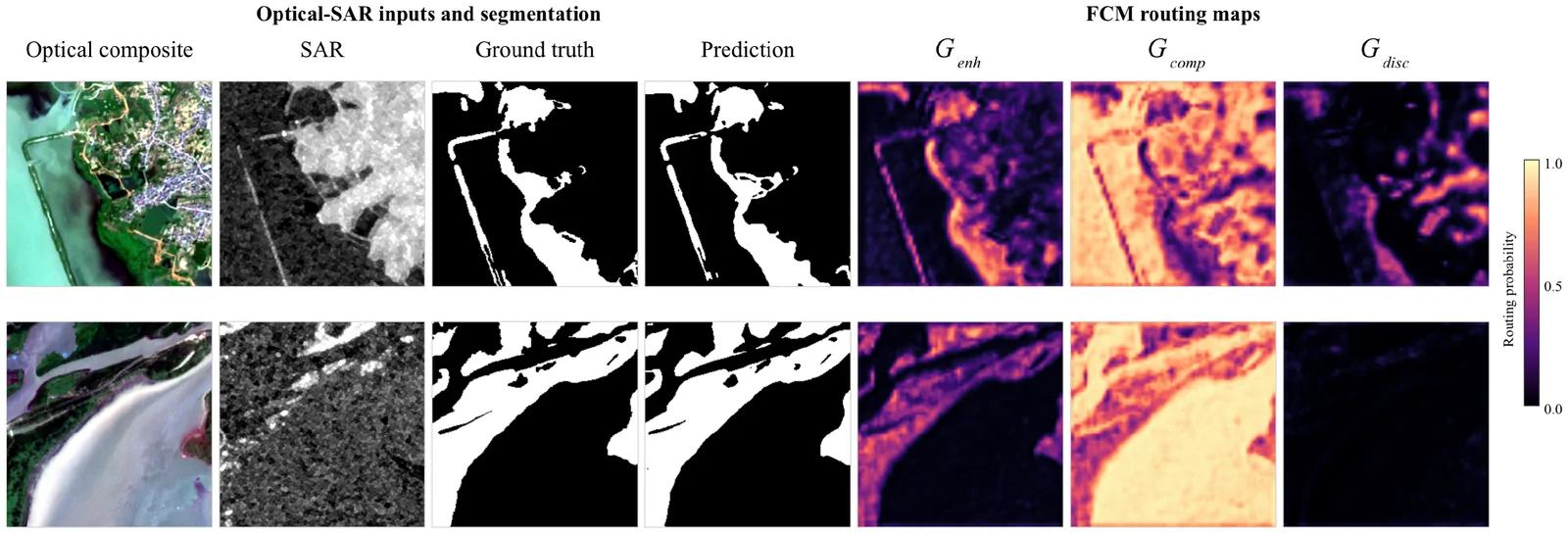
FCM routing maps for two CAU-Flood image pairs.

**FCM routing visualization.** To examine internal behavior not captured by the prediction-only ablation results, Figure 11 presents two representative CAU-Flood cases at FCM1, the highest-resolution fusion stage.

The first four columns of Figure 11 present the optical composite, SAR image, ground truth, and prediction. The remaining columns present $G_{\mathrm{enh}}$, $G_{\mathrm{comp}}$, and $G_{\mathrm{disc}}$ (the discrepancy-suppression gate) on a common probability scale from 0 to 1. In both cases, the enhancement response is concentrated around structured transitions, whereas complementation covers broader areas. The discrepancy response is localized in the first case and nearly absent in the second, indicating that the three routes vary spatially rather than acting as fixed global weights.

The gate names refer to operations learned by FCM rather than supervised physical-region labels. Because CAU-Flood does not provide pixel-wise annotations for cloud, SAR speckle, or cross-modal relation type, the maps reveal spatial variation in the routing responses but cannot establish a one-to-one correspondence between any gate and a specific degradation.

We assess modality dependence from two complementary perspectives. The Optical-only and SAR-only baselines are trained and evaluated with the indicated sensor alone, measuring the information available when the model is optimized for a single observation. Under the principal CAU-Flood pairing, Optical-only lacks the post-disaster observation, whereas SAR-only lacks the pre-disaster reference; neither baseline therefore retains the complete bitemporal evidence required to identify inundation change. By contrast, CISRMamba and its w/o-RFI variant are trained with paired inputs, and four controlled interventions are introduced only at inference. Drop MSI and Drop SAR replace the corresponding image with its channel-wise mean, removing spatial information without changing the input dimensionality. Cloud-50% masks a contiguous half of the MSI, whereas Speckle-L2 applies unit-mean multiplicative Gamma noise with L=2 to SAR.

The results are summarized in Table 5.

The Optical-only and SAR-only baselines achieve 59.59% and 61.59% mIoU, respectively, whereas the paired optical–SAR model reaches 91.53%. This difference is consistent with the bitemporal formulation: Optical-only lacks post-disaster evidence of the flood event, while SAR-only lacks the pre-disaster reference needed to distinguish newly inundated areas from persistent water. The single-modality and Drop experiments represent different operating conditions. A single-modality model adapts its feature extractor and prediction function to one sensor throughout training; Drop MSI and Drop SAR instead expose a paired-input model to an unseen mean-filled stream at inference. Thus, the Drop scores measure sensitivity to unexpected modality loss, whereas the single-modality scores reflect adaptation to a fixed one-sensor setting. In the matched comparison, RFI improves mIoU by 1.01, 0.75, 1.21, 1.74, and 11.13 percentage points under Clean, Drop MSI, Drop SAR, Cloud-50%, and Speckle-L2, respectively. The improvement is observed under every evaluated condition and is largest under SAR speckle degradation (11.13 percentage points). These results are consistent with more effective cross-modal information utilization, although this interpretation is limited to the tested interventions.

The ablation results quantify changes in segmentation performance, while the FCM maps directly show spatial variation in the routing responses. Accordingly, modality suppression, boundary-adaptive scanning, and high-frequency detail recovery are interpreted as behaviors consistent with the intended module designs rather than as directly verified internal causal mechanisms.

### 4.3. Comparison of Different Backbones

To evaluate the impact of backbone selection, the encoder is replaced with several alternatives while FCM, RFI, and SRB remain unchanged. As presented in Table 6, ResNet50, Swin-T, and SegMAN-B [51] achieve mIoU scores of 90.75%, 90.89%, and 90.93%, respectively.

Removing the SS2D route reduces mIoU to 91.04%, whereas removing the wavelet reconstruction route reduces it to 90.88%, corresponding to decreases of 0.49 and 0.65 percentage points relative to the complete MobileMamba encoder. The SS2D route combines long-range spatial propagation with input-conditioned direction weighting, while the wavelet route preserves complementary high-frequency information. Because direction weighting is embedded within SS2D, the w/o SS2D variant evaluates the complete route rather than the independent effect of directional weighting. These ablations isolate the contribution of each route but do not quantify their interaction.

### 4.4. Summary of Results

On the CAU-Flood dataset, CISRMamba achieves a mean intersection over union (mIoU) of 91.53% and surpasses the CNN, Transformer, and standard Mamba baselines across all five evaluation metrics. Table 3 further indicates that the enhanced MobileMamba encoder with DSR attains this performance with 53.51 million parameters and 13.95 GMACs.

On the Wuhan dataset, CISRMamba achieves the highest mIoU of 58.67% and demonstrates the most favorable precision–recall trade-off among the evaluated methods. Taken together, the ablation and backbone comparisons establish the performance contribution of each component under the evaluated settings. The observed changes are consistent with the intended roles of DSR in adapting the scan geometry, FCM and RFI in optical–SAR interaction and integration, and SRB in refining high-frequency detail. However, these results do not by themselves directly verify the corresponding internal mechanisms.

### 4.5. Limitations and Challenges

Several challenges must be addressed before CISRMamba can be deployed in broader operational contexts.

- **Computational footprint.** While CISRMamba is more efficient than many recent architectures, the computational requirements of 53.51 million parameters and 13.95 GMACs remain high for ultra-low-power platforms such as nanosatellites or small UAVs. Deployment in time-critical response scenarios will likely require hardware-aware pruning, quantization, or kernel-level optimization.
- **Paired bitemporal input without explicit temporal modeling.** The current model processes a single optical–SAR pair rather than a multi-temporal sequence. In CAU-Flood, this pair primarily combines a pre-disaster optical reference with a post-disaster SAR observation. However, because CISRMamba receives neither acquisition timestamps nor the inter-image interval, it cannot explicitly distinguish sensor disagreement from seasonal land-cover change, event evolution, or residual misregistration. Its performance has not been established for arbitrary temporal gaps or reversed acquisition order. Tracking flood expansion and recession would require explicitly timestamped multi-temporal observations.
- **Geographic generalization.** Although CAU-Flood includes several flood-prone regions, its preprocessing excludes pixels with slopes above 5°, resulting in a benchmark dominated by low-relief floodplains. The performance of CISRMamba in mountainous terrain, arid regions, or areas with distinct land-cover patterns has yet to be evaluated.
- **Residual errors in complex scenes.** Figure 8 and Figure 9 show remaining errors around small water fragments between buildings and around surfaces with similar appearances, including radar shadow, layover, smooth wet soil, and shallow or vegetation-covered water. A single paired optical–SAR observation may not contain enough evidence to distinguish these cases from open water. The network learns statistical associations from the training data rather than applying explicit hydrological rules; as a result, ambiguous pixels may remain unresolved when neither spectral information nor backscatter provides a clear cue.

### 4.6. Implications and Future Work

Deployment is one priority for future research. The selective scan mechanism will be adapted to lightweight processors with the aim of reducing inference latency on embedded hardware. A second direction is adaptive computation: simple open-water scenes could be processed with fewer operations, while dense urban inundation scenes would receive more detailed analysis.

Future work will extend CISRMamba from the current paired bitemporal setting to multi-temporal flood analysis with explicit timestamps. Continuous observations or additional same-sensor pre- and post-disaster references could help model event evolution and distinguish permanent or seasonal water from newly emerging floodwater.

Geographic generalization remains an open issue. Future evaluations should include a wider range of climate zones, land-cover patterns, and terrain types to more effectively assess domain shifts in operational contexts.

## 5. Conclusions

This paper presents CISRMamba, a multimodal state space model designed for optical–SAR flood mapping. It targets two main bottlenecks in current fusion approaches: modality collapse caused by optical–SAR distribution shifts and the fixed scanning patterns of standard state space models on irregular flood boundaries. More broadly, CISRMamba conceptualizes flood mapping as a heterogeneous multi-sensor fusion problem, in which optical spectral cues and SAR backscattering responses are processed jointly but not uniformly. DSR is designed to adapt the scan geometry to irregular spatial structures, while FCM routes cross-modal information through discrepancy suppression, complementation, and enhancement. RFI uses a difference-guided residual to preserve modality-specific information during integration, and SRB refines high-frequency detail through spectral-domain reconstruction. Experimental results demonstrate 91.53% mIoU on CAU-Flood and 58.67% mIoU on Wuhan, with consistent improvements over CNN, Transformer, and Mamba baselines. Together with the component ablations and FCM routing maps, these results support the contribution of the proposed design to segmentation performance on the evaluated benchmarks. The measured evidence comprises prediction accuracy, ablation differences, and spatial routing responses; the specific module behaviors remain interpretations consistent with the proposed mechanisms rather than directly observed causal processes. Future research will extend the spatial fusion design to multi-temporal flood monitoring.

## Figures and Tables

**Table 1:** Performance comparison on CAU-Flood. Bold values indicate the best result, and underlined values indicate the second-best result.

Model	mIoU (%)	Acc (%)	F1 (%)	Precision (%)	Recall (%)
HANet	85.41	96.76	91.33	93.30	89.44
RFANet	86.19	96.56	91.94	92.62	91.26
DeepLabV3+	87.65	96.52	92.44	93.76	91.14
UNet++	88.33	96.89	93.61	93.14	94.09
SemTFormer	88.62	96.84	93.03	94.23	91.86
CMCDNet	89.67	97.22	94.27	93.74	94.80
CCDCNet	89.85	97.39	94.52	94.96	94.09
UCDFormer	90.00	97.38	94.57	94.25	94.90
MambaSeg	90.18	97.45	94.68	94.76	94.61
SD-Mamba	90.27	97.47	94.75	94.72	94.77
**Ours**	**91.53**	**97.81**	**95.47**	**95.23**	**95.73**

**Table 2:** Quantitative results on the Wuhan dataset. Bold values indicate the best result, and underlined values indicate the second-best result.

Model	mIoU (%)	Acc (%)	F1 (%)	Precision (%)	Recall (%)
HANet	50.14	85.13	61.36	65.52	57.70
SemTFormer	51.65	85.95	63.27	68.81	58.56
RFANet	52.05	85.12	63.29	66.59	60.30
CCDCNet	52.42	85.14	62.80	67.50	58.71
MambaSeg	52.88	85.15	63.50	68.10	59.48
DeepLabV3+	54.80	85.79	67.07	67.21	66.93
CMCDNet	55.06	86.60	65.61	68.79	63.77
UCDFormer	55.19	87.06	65.50	**73.40**	62.59
SD-Mamba	55.23	86.58	68.32	72.64	64.48
UNet++	56.02	86.92	66.49	71.75	63.96
**Ours**	**58.67**	**87.67**	**70.65**	72.89	**68.56**

**Table 3:** Component ablation results for the proposed method. “✓” denotes that a module is present, and “×” denotes that it is absent. For the performance metrics, bold values indicate the best result, and underlined values indicate the second-best result. DSR is listed alongside RFI, FCM, and SRB.

DSR	RFI	FCM	SRB	mIoU (%)	Acc (%)	F1 (%)	Precision (%)	Recall (%)	Params (M)	GMACs
✓	×	×	×	89.51	97.14	92.99	93.85	92.15	52.83	12.93
✓	✓	×	×	90.39	97.35	94.23	94.02	94.44	53.17	13.39
✓	×	✓	×	90.52	97.52	94.53	94.41	94.65	53.00	13.08
✓	×	×	✓	89.98	97.28	93.98	93.72	94.24	53.00	13.35
✓	✓	✓	×	91.05	97.71	95.18	**95.70**	94.67	53.34	13.53
×	✓	✓	✓	91.11	97.69	94.98	94.86	95.11	53.46	13.93
✓	✓	✓	✓	**91.53**	**97.81**	**95.47**	95.23	**95.73**	53.51	13.95

**Table 4:** Boundary-specific ablation of DSR and SRB on CAU-Flood. BF@*r* denotes the boundary F1 score at an *r*-pixel matching tolerance; Boundary IoU is computed within a fixed 3-pixel boundary band. Bold values indicate the best result in each metric, and underlined values indicate the second-best result.

DSR	SRB	BF@1 (%)	BF@2 (%)	BF@3 (%)	Boundary IoU (%)
×	×	72.01	81.08	84.84	64.20
✓	×	74.10	83.20	86.11	66.97
×	✓	74.81	82.70	86.56	67.24
✓	✓	**76.50**	**84.52**	**87.71**	**68.25**

Note: ✓ and × indicate that the corresponding module is included or excluded, respectively. FCM, RFI, and all other components are retained in every configuration.

**Table 5:** Modality-specific and degradation-based evaluation on CAU-Flood. Bold values indicate the best result in each evaluated condition, and underlined values indicate the second-best result.

Model	Input	Clean	Drop MSI	Drop SAR	Cloud-50%	Speckle-L2
Optical-only	Pre-MSI	59.59	–	–	–	–
SAR-only	Post-SAR	61.59	–	–	–	–
w/o RFI	MSI + SAR	90.52	42.43	51.49	50.35	64.60
CISRMamba	MSI + SAR	**91.53**	**43.18**	**52.70**	**52.09**	**75.73**

Note: Pre-MSI and Post-SAR denote the pre-disaster multispectral and post-disaster SAR observations, respectively. All values are mIoU (%); “–” denotes a condition not evaluated for a single-modality baseline.

**Table 6:** Quantitative comparison of different backbones on the CAU-Flood dataset. Bold values indicate the best result, and underlined values indicate the second-best result.

Backbone	mIoU (%)	Acc (%)	F1 (%)	Precision (%)	Recall (%)
ResNet50	90.75	97.47	94.35	94.12	94.58
Swin-T	90.89	97.55	94.72	94.55	94.89
SegMAN-B	90.93	97.66	94.87	94.70	95.04
w/o SS2D	91.04	97.69	94.77	94.86	94.69
w/o Wavelet	90.88	97.65	94.50	95.09	93.91
**Ours**	**91.53**	**97.81**	**95.47**	**95.23**	**95.73**

Note: The “w/o SS2D” and “w/o Wavelet” rows denote MobileMamba encoder variants without the SS2D route and the wavelet reconstruction route, respectively.

## Data Availability

The datasets used in this study are publicly available. CAU-Flood is hosted in the CMCDNet GitHub repository at https://github.com/CAU-HE/CMCDNet (accessed on 13 August 2026) and is described in [25]. The Wuhan dataset is available from the GitHub repository associated with A Domain Adaptation Neural Network for Change Detection with Heterogeneous Optical and SAR Remote Sensing Images at https://github.com/GeoZcx/A-Domain-Adaption-Neural-Network-for-Change-Detection-with-Heterogeneous-Optical-and-SAR-Remote-Sens (accessed on 13 August 2026) and is described in [42]. The source code for the proposed method is available in the CISRMamba repository at https://github.com/fffhhhrrr333/CISRMamba (accessed on 13 August 2026).

## Abbreviations

The following abbreviations are used in this manuscript:

CVA	Change Vector Analysis
DEM	Digital Elevation Model
DSR	Deformable Scan Router
DWT	Discrete Wavelet Transform
FCM	Feature Complementary Module
FCN	Fully Convolutional Network
IDWT	Inverse Discrete Wavelet Transform
MSI	Multispectral Instrument
NDWI	Normalized Difference Water Index
RFI	Refined Feature Integration
SAR	Synthetic Aperture Radar
SRB	Spectral Refinement Block
SS2D	Two-Dimensional Selective Scan
SVM	Support Vector Machine
